# Activation patterns in male and female forebrain circuitries during food consumption under novelty

**DOI:** 10.21203/rs.3.rs-3328570/v1

**Published:** 2023-09-11

**Authors:** Eliza M. Greiner, Mary E. Witt, Stephanie J. Moran, Gorica D. Petrovich

**Affiliations:** Boston College; Boston College; Boston College; Boston College

**Keywords:** novelty, consumption, sex differences

## Abstract

The influence of novelty on feeding behavior is significant and can override both homeostatic and hedonic drives due to the uncertainty of potential danger. Previous work found that novel food hypophagia is enhanced in a novel environment and that males habituate faster than females. The current study’s aim was to identify the neural substrates of separate effects of food and context novelty. Adult male and female rats were tested for consumption of a novel or family food in either a familiar or in a novel context. Test-induced Fos expression was measured in the amygdalar, thalamic, striatal, and prefrontal cortex regions that are important for appetitive responding, contextual processing, and reward motivation. Food and context novelty induced strikingly different activation patterns. Novel context induced Fos robustly in almost every region analyzed, including the central (CEA) and basolateral complex nuclei of the amygdala, the thalamic paraventricular (PVT) and reuniens nuclei, the nucleus accumbens (ACB), the medial prefrontal cortex prelimbic and infralimbic areas, and the dorsal agranular insular cortex (AI). Novel food induced Fos in a few select regions: the CEA, anterior basomedial nucleus of the amygdala, anterior PVT, and posterior AI. There were also sex differences in activation patterns. The capsular and lateral CEA had greater activation for male groups and the anterior PVT, ACB ventral core and shell had greater activation for female groups. These activation patterns and correlations between regions, suggest that distinct functional circuitries control feeding behavior when food is novel and when eating occurs in a novel environment.

## Introduction

Novel stimuli are initially treated with weariness or avoidance. This is an adaptive response that allows for evaluation of danger or risk posed by the novel stimulus. However, when these avoidant behaviors become persistent, they can become maladaptive and result in the development of psychopathology. Restrictive eating is a core symptom in Anorexia Nervosa and Avoidant/Restrictive Food Intake Disorder (Treasure et al. 2020; Zimmerman and Fisher 2017).

Interactions with new foods are critical because of the potential risk of illness after consumption. A common behavioral reaction to novel foods is *taste neophobia*. In animals, taste neophobia is defined as lower consumption of a new taste during initial exposures compared to when the taste is familiar and food is considered safe (Lin, et al., 2012).

Novelty of the environment also powerfully impacts feeding behavior. Rodents have been shown to have longer latencies to consume food in a novel open field (for review see Ramaker & Dulawa, 2017). However, prior preparations, traditionally, only studied males. Recent work that compared the impact of novel environments on feeding in males and females found sex differences and more pronounced effects in females (Greiner & Petrovich, 2020; De Oliveira Sergio et al 2021). Females had longer latency to approach food in a brightly lit open arena and lower consumption than males (De Oliveira Sergio et al 2021). Male and female rats that were given a choice of novel and familiar foods in novel or familiar environments, suppressed feeding in a new context in a sex dependent manner (Greiner & Petrovich, 2020). In a novel context, males habituated to eating a novel food faster than females, who showed suppressed consumption throughout testing (Greiner & Petrovich, 2020). The prolonged suppression in females may be relevant to sex differences in avoidant behaviors (Sheynin et al., 2014) and the development of Anorexia Nervosa and Avoidant/Restrictive Food Intake Disorder (Treasure et al., 2020; Zimmerman & Fisher 2017). However, there is a significant gap in our knowledge about the neural substrates underlying novelty effects on feeding in males and females.

The current study systematically examined Fos induction in key forebrain regions during the consumption of novel or familiar foods in novel or familiar environments and compared patterns in male and female rats. While the underlying neural circuity is largely unknown, a specific subset of interconnected cortical, thalamic, striatal, and amygdalar areas are strong candidates for mediating consumption during novelty exposure. The amygdala is essential for emotional learning and memory consolidation, and the basolateral complex nuclei and the central nucleus (CEA) were examined in the current study because they play integral roles in appetitive behavior (reviewed in Cole et al., 2013). The CEA and the basolateral complex nuclei are activated by novel food (Koh et al., 2003; Lin et al., 2012), and bilateral lesions of the CEA in rats eliminated feeding inhibition under fear (Petrovich et al., 2009), while lesions to the basolateral complex nuclei lessened neophobic reactions to novel tastes in familiar environments (Nachman & Ashe, 1974; Lin et al., 2009).

Two midline thalamic nuclei that are important for appropriate regulation of avoidance behavior were analyzed, the paraventricular nucleus of the thalamus (PVT) and the nucleus reuniens (RE). The PVT is interconnected with the CEA and basolateral complex nuclei and is known for the regulation of food consumption and body weight (Li & Kirouac, 2008; Bhatnagar & Dallman, 1999; Petrovich, 2018). The PVT was a part of the recruited network for contextual mediation of appetitive behavior—renewal of responding to food cues after extinction (Anderson & Petrovich, 2017). The PVT has also been shown to regulate the motivation to eat in novel environments. Optogenetic activation of the anterior PVT increased feeding in a novel open field (Cheng et al., 2018). The RE is necessary for appropriate regulation of avoidance behavior (Linley et al., 2020). Additionally, it serves as a major link between the medial prefrontal cortex (mPFC) and the hippocampal formation (McKenna & Vertes, 2004), which is relevant for contextual processing of novel environments.

The medial prefrontal cortex (mPFC) is necessary in decision making, particularly in the calculation of risk versus reward (Bechara & Damasio, 2005). It is also critical in regulation of food consumption under cognitive control (learned cues) (Petrovich et al., 2007; Cole et al., 2020) and via opioid stimulation (Mena, Sadeghian, Baldo 2011). The infralimbic (ILA) and prelimbic (PL) subregions of the mPFC, receive extensive and highly organized projections from the basolateral complex nuclei of the amygdala (Reppucci & Petrovich, 2016), which could impact mPFC processing during decision making. In turn, the mPFC projects directly to both the CEA (Hurley et al., 1991) and the basolateral complex nuclei (Gabbott et al., 2005) and these connections have been shown to control behavioral outputs (Quirk et al., 2003). Connections between the vmPFC and BLA are particularly important for reward restraint (Ishikawa et al., 2019), to prevent animals from engaging in appetitive behaviors when there is a potential risk.

Stimulation of the axon terminals from D1-type dopamine receptor expressing mPFC neurons to the basolateral nucleus of the amygdala (BLA) neurons was shown to increase feeding (Land et al., 2014). The mPFC is also a key region of interest for sex differences in the regulation of feeding behavior in a novel context. The mPFC was engaged differently in males and females during context-induced renewal of responding to food cues (Anderson & Petrovich, 2017), and during feeding tests when hunger and fear compete (Reppucci & Petrovich 2018).

The role of the nucleus accumbens (ACB) in appetitive motivation is well known (for reviews see Wise, 2000; Kelley, 2004; Salamone, 1994; Morales & Berridge, 2020). The ACB is interconnected with the PVT (Parsons et al., 2007; Dong et al., 2017), mPFC (Sesack et al., 1989; Groenewegen et al., 1999), and BLA (Christie et al., 1987; Brog et al., 1993). Additionally, the ACB shell (ACBsh) contains hedonic hotspots that drive the motivation to eat palatable foods (Castro et al., 2016; Thompson & Swanson, 2010).

The agranular insular cortex (AI) processes visceral and taste information (Gogolla 2017) and is interconnected with the PVT, the CEA and basolateral complex nuclei, and mPFC (Li and Kirouac, 2012; McDonald 1998; Shi & Cassell, 1998). Lesions to the AI blocked conditioned taste aversion behavior (Cubero, Thiele, & Bernstein, 1999), while novel taste induced Fos expression in the AI, similar to the patterns in the CEA (Koh, Wilkins, & Bernstein, 2003).

Here, we determined Fos induction patterns within the above identified areas of interest, in order to outline the neural networks that mediate novelty effects on food consumption and sex differences within these networks. For clarity of neural analysis, rats were given access to only one food during testing, either novel or familiar, and they were tested in either a novel or familiar context. This paradigm design allowed us to separately analyze the effects of sex, context, and food type on consumption and neural activation.

## Materials & Methods

### Subjects

Adult male (n = 32) and female (n = 32) Long Evans rats (Charles River Laboratories; Portage, MI), that weighed 225–250g upon arrival, were individually housed and maintained on a 12-hour light/dark cycle (lights on 06:00). Males and females were housed in the same colony room on separate shelves. After arrival, subjects were allowed one week to acclimate to the colony housing room before behavioral procedures began, during which they had *ad libitum* access to water and standard Rat chow (Purina Lab Diet Prolab RMH 3000; 3.47 kcal/g; 26% protein, 15% fat, 59% carbohydrates), and were handled daily. All housing and testing procedures were in compliance with the National Institutes of Health Guidelines for Care and Use of Laboratory Animals and approved by the Boston College Institutional Animal Care and Use Committee.

### Apparatus

Half of the animals were tested in a familiar environment (their housing cages; Home Cage) and the other half were tested in a novel environment (behavioral chamber; plexiglass box (30×28×30cm) with grid flooring and a recessed port (3.2 ×4.2 cm) on one wall; Coulbourn Instruments). Each chamber was enclosed in monolithic rigid foam box. Food was presented in a ceramic bowl.

### Behavioral Testing Procedure

Male and female rats were tested for consumption of either a novel or a familiar food in either a novel or familiar environment and, after testing, the brain tissue was collected for later processing. There were eight groups in order to test the effects of sex, testing context, and food presented: Females given a familiar food in a familiar context, males given a familiar food in a familiar context, females given a novel food in a familiar context, males given a novel food in a familiar context, females given a familiar food in a novel context, males given a familiar food in a novel context, females given a novel food in a novel context, and males given a novel food in a novel context. All groups underwent one 30-minute testing session. Prior to testing all rats were food deprived for 20 hours. For the test, each rat was presented with a ceramic bowl that contained either 15g of a familiar food (Rat Chow) or 15g of a novel food (TestDiet (TD) pellets; 3.4 kcal/g; 21% protein, 13% fat, 67% carbohydrate; 5TUL 45mg).

All rats were habituated to transport to the conditioning chamber room, as well as to the ceramic bowls, at least 24 hours prior to testing. The weight of all foods was measured following the end of testing to determine how much was consumed. Body weights for all rats were taken in the morning of test day. Average body weights were calculated for each group. All consumption data is presented as a number of grams consumed per 100 grams of body weight.

### Histological Procedures

Rats were perfused 90 minutes after start of testing and brains were harvested. Rats were briefly anesthetized with isoflurane (5%; Baxter Healthcare Corporation, Deerfield, IL), and then deeply anesthetized with an intraperitoneal injection of tribromoethanol (375 mg/kg; Sigma-Aldrich, St. Louis, MO). Rats were then transcardially perfused with 0.9% saline followed by 4% paraformaldehyde in 0.1 M borate buffer. Brains were extracted and post-fixed overnight in a solution of 12% sucrose dissolved in the perfusion liquid, then rapidly frozen in hexanes cooled in dry ice and stored at − 80°C. Brains were sliced in 30-μm sections using a sliding microtome and collected into four adjacent series.

The first series was stained using standard immunohistochemical procedures for visualization of Fos. Free-floating tissue sections were incubated in a blocking solution for 1 h at room temperature to minimize nonspecific binding. The blocking solution contained 0.02M potassium phosphate-buffered saline (KPBS), 0.3% Triton X-100 (Sigma-Aldrich), 2% normal goat serum (S-1000; Vector Laboratories, Burlingame, CA), and 10% non-fat milk (M-0841; LabScientific, Livingston, New Jersey). Then, the tissue was incubated with the primary antibody, anti-*c-fos* raised in rabbit (1:5,000, ABE457, EMD Millipore, Billercia, MA; or 1:5,000, 226 003, Synaptic Systems, Gottingen, Germany; the use of each antibody was counterbalanced across training conditions) in the blocking solution for 72 h at 4°C. The tissue was rinsed in KPBS then incubated with the secondary antibody, biotinylated goat anti-rabbit IgG (1:500; BA-1000; Vector Laboratories) in the blocking solution for 45 min. Subsequently, the tissue was rinsed in KPBS then reacted with avidin–biotin complex (ABC solution; PK-6100; Vector Laboratories) for 45 min. To improve specific binding, this was followed by rinses in KPBS, a second 30 min incubation in the secondary antibody solution, rinses in KPBS, a second 30 min incubation in the ABC solution, and additional rinses in KPBS. To produce a color reaction, the tissue was incubated in a diaminobenzidine solution (SK-4100; Vector Laboratories) for 1–2 min with constant, manual agitation. Stained tissue was then mounted onto SuperFrost Plus slides (Fisher Scientific, Pittsburgh, PA) and air-dried, followed by drying in an oven at 45°C overnight. Tissue was then dehydrated through graded alcohols, cleared in xylenes, and coverslipped with DPX (13512; Electron Microscopy Sciences, Hatfield, PA).

The second series was collected into KPBS solution, mounted onto gelatin-subbed slides, and stained with thionin for identification of cytoarchitectonic borders of brain structures, as defined in Swanson’s rat brain atlas (Swanson 2018). The remaining series were collected into trays containing a cryoprotectant solution (0.025 M sodium phosphate buffer with 30% ethylene glycol and 20% glycerol) and stored at − 20°C for later use. Brain perfusions, collection, slicing, and length of storage were counterbalanced across training conditions.

### Image Acquisition & Analysis

Images of stained tissue were acquired with an Olympus BX51 light microscope at 10X and attached Olympus DP74 camera using DP2-BSW software (Olympus America Inc, Center Valley, PA). Using the ImageJ software program (NIH), borders for regions of interest were drawn onto the image of the thionin-stained tissue, and then transposed to the image of the adjacent immunohistochemically-stained tissue to allow for semi-automated counting of Fos-positive neurons based on size and circularity measures. Identification of regions and borders for analysis were determined based on the Swanson rat brain atlas (Swanson, 2018). Representative atlas levels and distances from bregma for each analyzed region is documented in the table below (Table 1). Analysis was conducted across the rostro-caudal extent of each subregion of the CEA: capsular (CEAc), lateral (CEAl), and medial (CEAm). Within the basolateral complex, each nucleus was analyzed: anterior basolateral nucleus (BLAa), posterior basolateral nucleus (BLAp), anterior basomedial nucleus (BMAa), posterior basomedial nucleus (BMAp), and the lateral amygdala (LA). Analysis for ACB was conducted for each subregion: core (ACBc), dorsal shell (ACBdsh), and ventral shell (ACBvsh). The PVT was analyzed at a representative anterior (aPVT) and posterior (pPVT) level. Analysis of RE was conducted on a single representative level. The subregions of the mPFC (PL and ILA) were each analyzed on a separate representative level. For the AI analysis was conducted on a representative level for the dorsal AI (AId) and posterior AI (AIp). Bilateral images were acquired for all regions, except for the PVT and RE, where both sides were acquired in a single image. Images were analyzed for each region of interest; counts from left and right hemispheres were summed for each rat to calculate the total number of Fos-positive neurons per region.

### Statistical Analysis

Following arrival, males gained weight faster than females, resulting in body weight differences by the time of testing. Therefore, all consumption results are reported as grams consumed per 100 grams of body weight ([food consumed(g)/body weight(g)]X100).

Consumption results were analyzed using a between-subjects 3-way univariate ANOVA for food type, sex, and testing context. Analysis of subregions and anatomical levels of interest for each region were analyzed using 3-way multivariate ANOVAs for food type, sex, and context. All significant interactions were followed by Bonferroni *post hoc* analyses.

Bivariate Pearson correlation analysis were conducted within each testing group to assess the relationship of Fos induction between each subregion analyzed. For this analysis, the CEA was collapsed across the two anterior (levels 25 & 26) and posterior (levels 27 & 28) anatomical levels analyzed for each subregion (anterior, aCEAm, aCEAl, & aCEAc; posterior, pCEAm, pCEAl, & pCEAc). A value of p < 0.05 was considered significant for all analyses, except for *post-hoc* analyses in which Bonferroni adjusted alpha level was used (p = 0.05/3 = 0.017). A value of p < 0.09 was considered a trend towards significance.

## Results

### Food Consumption

**Figure 1:**
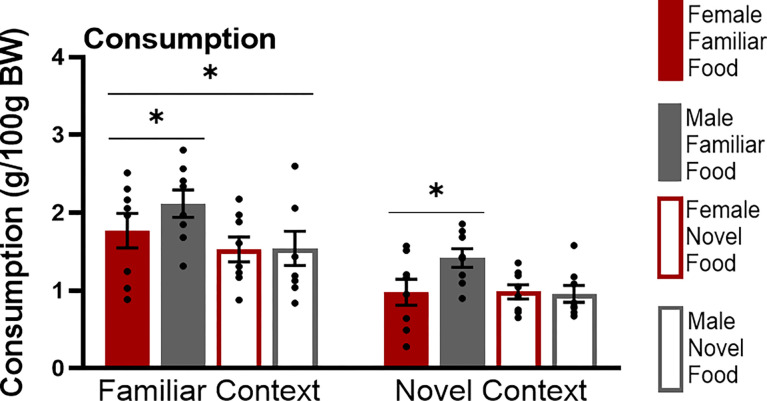
Food consumption test. The graphs show the amounts of each food that subjects in each testing condition consumed, expressed as grams per 100 grams of their body weight (BW). Asterisks indicate p<0.05.

Consumption during testing differed based on food type and context familiarity (Figure 1). Male and female rats given a familiar food ate more than male and female rats given a novel food (F(1,52) = 7.509 p = 0.008) and groups tested in a familiar context had greater consumption compared to groups tested in a novel context (F(1,52)=26.767 p<0.001) regardless of food type. Male and female groups were similar (F(1,52)=2.313 p=0.13) and there were no interactions of any factor (sex by food F(1,52)=2.598 p=0.11, sex by context F(1,52)=0.003 p=0.96, food by context F(1,52)=0.356 p=0.5, sex by food by context F(1,52)=0.066 p=0.79).

### Fos Induction

#### Central Nucleus of the Amygdala

**Figure 2:**
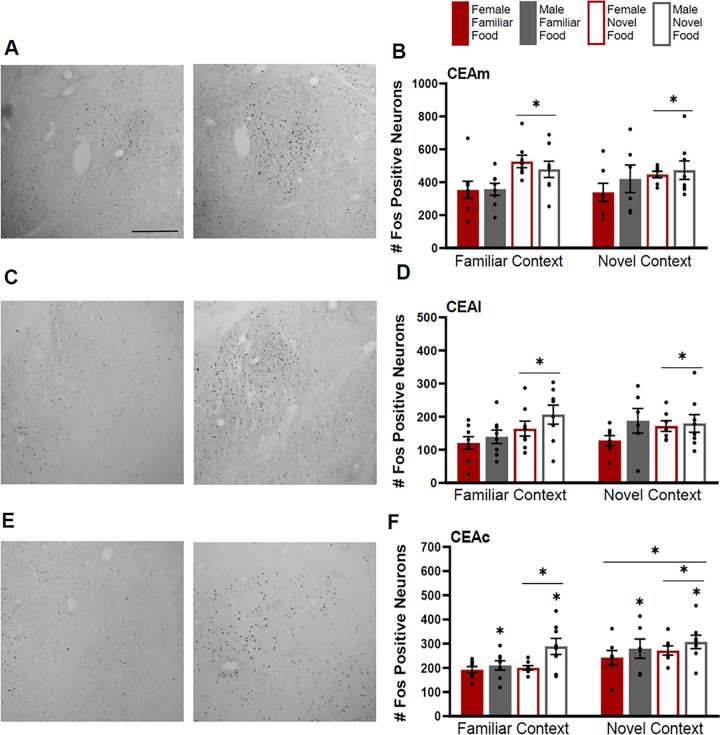
Fos induction in the medial (CEAm), lateral (CEAl), and capsular (CEAc) subregions of the central nucleus of the amygdala. **A)** Tissue images stained for Fos of the CEAm (atlas level 26, right side) for a female tested in a familiar context given a familiar food (left image) and a female tested in a familiar context given a novel food (right image). **B)** Fos induction for each testing condition in the CEAm. **C)** Tissue images stained for Fos of the CEAl (atlas level 28, right side) for a male tested in a familiar context given a familiar food (left image) and a male tested in a familiar context given a novel food (right image). **D)** Fos induction for each testing condition in the CEAl. **E)** Tissue images stained for Fos of the CEAc (atlas level 27, right side) for familiar context tested female given a novel food (left image) and a novel context tested female given a novel food (right image). **F)** Fos induction for each testing condition in the CEAc. Asterisks indicate p<0.05. Scale bar in upper left image=500um.

Each CEA subregion (medial, lateral, and capsular) had similar increase in Fos induction in rats that were given novel food (Figure 2 B–D) (CEAm F(1,52)=10.196, p<0.01; CEAl, F(1,52)=4.658, p=0.036; CEAc, F(1,52)=4.166, p=0.046). In addition, in the CEAc, all rats tested in a novel context, had more Fos positive neurons compared to those tested in a familiar context, and the induction was overall higher for males compared to females (Figure 2 D) (context: F(1,52)=8.926, p<0.01; sex: F(1,52)=6.449, p=0.014). In the CEAm, there was a trend towards significance for context (F(1,52)=0.069, p=0.79) and no effect of sex (F(1,52)=0.198, p=0.66). In the CEAl, there was no effect for context (F(1,52)=0.287, p=0.59), but a trend towards significance for sex (F(1,52)=3.6, p=0.06).

Additional analysis examined CEA subregions across rostro-caudal levels. There was greater activation for rats tested in a novel context in CEAc at atlas level 25 and 27 (L25, F(1, 52)=5.676, p=0.021; L27, F(1, 52)=6.133, p=0.017) and in the CEAl at level 28 (F1, 52)=4.87, p=0.032). Of note, the males given a novel food in a familiar context had the greatest number of Fos positive neurons in the CEAl of L28 compared to all other groups. This was supported by a between-subjects interaction of context by food type by sex (F(1,52)=7.459, p=0.009) for the L28 CEAl. There were no effects of context for any other parts of the CEA at any rostro-caudal levels (p> 0.05 for all), except trends in L26-CEAl (F(1,52)=3.029, p=0.088) and L26-CEAc (F(1,52)=3.486, p=0.068).

There were additional between-subjects effects for food type where rats given a novel food had higher Fos induction compared to rats given a familiar food in the CEAm at level 27 and 28 (L27, F(1, 52)=25.096, p<0.001; L28, F(1, 52)=17.633, p<0.001) and for the CEAl at level 28 (F(1, 52)=4.664, p=0.035). There were no effects for food type in any other part of the CEA p> 0.05 for all), except for trends in L25-CEAm, F(1,52)=3.778, p=0.057 and L27-CEAl, F(1,52)=3.033, p=0.088).

#### Basolateral Nuclei of the Amygdala

**Figure 3:**
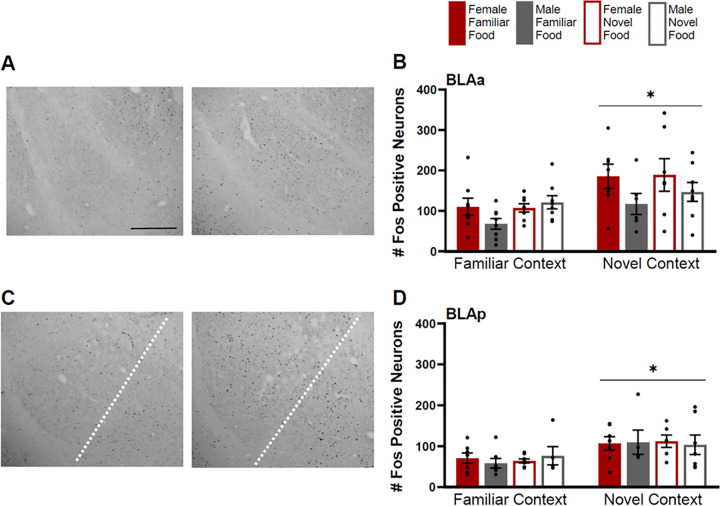
Fos induction in the anterior (BLAa) and posterior (BLAp) basolateral nuclei of the amygdala. **A)** Tissue images stained for Fos of the BLAa (atlas level 27, left side) for a female tested in a familiar context given a familiar food (left image) and a female tested in a familiar context given a novel food (right image). **B)** Fos induction for each testing condition in the BLAa. **C)** Tissue images stained for Fos of the BLAp (left of the dotted line; atlas level 30, left side) for a male tested in a familiar context given a familiar food (left image) and a male tested in a novel context given a familiar food (right image). Dotted line indicates a border between the BLAp and BMAp. **D)** Fos induction for each testing condition in the BLAp. Scale bar in upper left image=500um. Asterisks indicate p<0.05.

Fos induction in the BLAa and BLAp was greater for rats tested in a novel context than for rats tested in a familiar context (Figure 3) (BLAa: F(1,41)=12.534 p=0.001; BLAp: (F(1,41)=12.889 p=0.001)). There were no effects of food type (BLAa: F(1,41)=0.960 p=0.333; BLAp: F(1,41)=0.076 p=0.784) or sex (BLAa: F(1,41)=1.156 p=0.289; BLAp: F(1,41)=0.38 p=0.541) or any significant interactions in these regions (p>0.05 for all).

#### Basomedial Nuclei of the Amygdala

**Figure 4:**
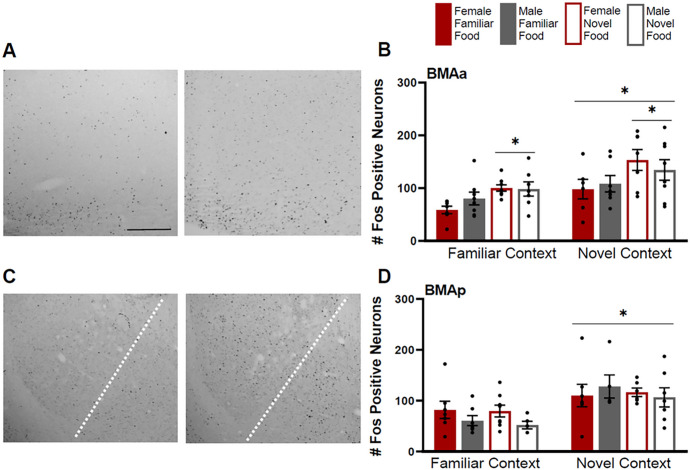
Fos induction in the anterior (BMAa) and posterior (BMAp) basomedial nuclei of the amygdala. **A)**Tissue images stained for Fos of the BMAa (atlas level 26, right side) for a female tested in a novel context given a familiar food (left image) and a female tested in a novel context given a novel food (right image). **B)** Fos induction for each testing condition in the BMAa. **C)** Tissue images stained for Fos of the BMAp (right of the dotted line; atlas level 30, left side) for a male tested in a familiar context given a familiar food (left image) and a male tested in a novel context given a familiar food (right image). Dotted line indicates a border between the BLAp and BMAp. **D)** Fos induction for each testing condition in the BMAp. Scale bar in upper left image=500um. Asterisks indicate p<0.05.

Fos induction in the BMAa was greater for rats tested in a novel context compared to rats tested in a familiar context (F(1,41)=9.408 p=0.004) and for rats given a novel food compared to those given a familiar food (F(1,41)=12.947 p=0.001) (Figure 4 A). There were no effects of sex (F(1,41)=0.592 p=0.446) or interactions (p>0.05 for all).

The BMAp had greater Fos induction for rats tested in a novel context (Figure 4 B) (F(1,41)=14.813 p<0.001) compared to rats tested in a familiar context, but had no effect of food type (F(1,41)=0.431 p=0.515), sex (F(1,41)=0.929 p=0.341), or any interactions (p>0.05 for all).

#### Lateral Nucleus of the Amygdala

**Figure 5:**
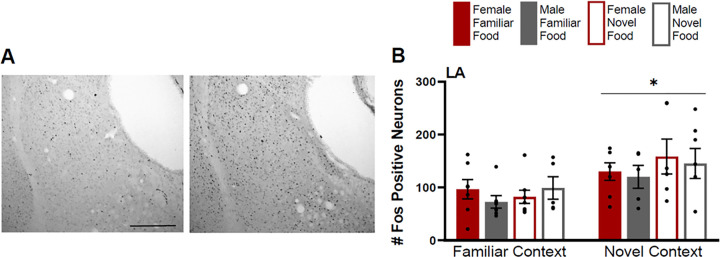
Fos induction in the lateral nucleus of the amygdala (LA). **A)** Tissue images stained for Fos of the LA (atlas level 30, left side) for a male tested in a familiar context given a familiar food (left image) and a male tested in a novel context given a familiar food (right image). **B)** Fos induction for each testing condition in the LA. Scale bar in left image=500um. Asterisks indicates p<0.05.

The Fos induction in the LA was greater for rats tested in a novel context (Figure 5) (F(1,41)=12.534 p=0.001) compared to rats tested in a familiar context, but there were no effects of food type (F(1,41)=0.1.108 p=0.299), sex (F(1,41)=0.242 p=0.625) or any interactions (p>0.05 for all).

#### Paraventricular Nucleus of the Thalamus

**Figure 6:**
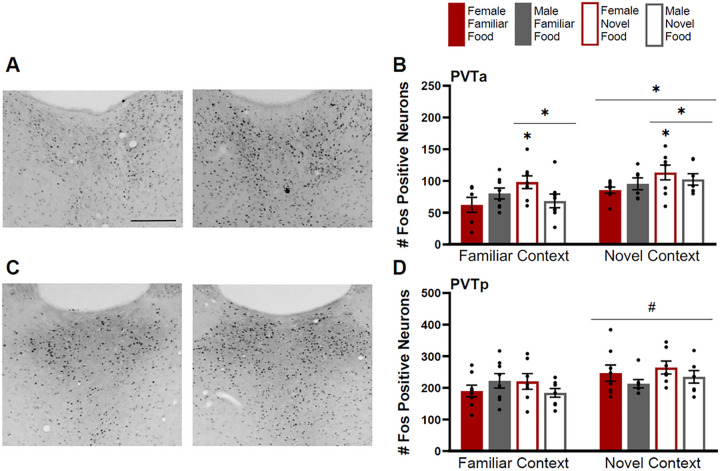
Fos induction in the anterior (PVTa) and posterior (PVTp) paraventricular nucleus of the thalamus. **A)** Tissue images stained for Fos of the PVTa (atlas level 26, midline) for a male tested in a familiar context given a novel food (left image) and a female tested in a familiar context given a novel food (right image**). B)** Fos induction for each testing condition in the PVTa. **C)** Tissue images stained for Fos of the PVTp (atlas level 31, midline) for a female tested in a familiar context given a familiar food (left image) and a female tested in a novel context given a familiar food (right image). **D)** Fos induction for each testing condition in the PVTp. Scale bar in upper left image=500um. Asterisks indicate p<0.05. Pound symbol indicates p=0.051.

The Fos induction in the PVTa was greater for rats tested in a novel context compared to those tested in a familiar context and those given a novel food compared to a familiar food (Figure 6 A–B). Additionally, females given a novel food had greater Fos induction than males given a novel food. There were significant main effects of food type (F(1,51)=4.149, p=0.047) and context (F(1,51)=9.355, p=0.004), but not sex (F(1,51)=0.157, p=0.69). There was a significant interaction of food type by sex (F(1,51)=5.605, p=0.22), but no other significant interactions (p>0.05 for all). A Bonferroni post hoc analysis revealed that among novel context tested animals, females had greater Fos induction than males (p=0.04)

The Fos induction in the PVTp was greater for rats tested in a novel context compared to a familiar context, however statistical analysis yielded results slightly above the level of significance for a main effect of context (F(1,51)=4.006, p=0.051) (Figure 6 C–D). There were no main effects of sex (F(1,51)=0.804, p=0.374), food type (F(1,51)=0.01, p=0.92) or interactions (p>0.05 for all).

#### Nucleus Reuniens of the Thalamus

**Figure 7:**
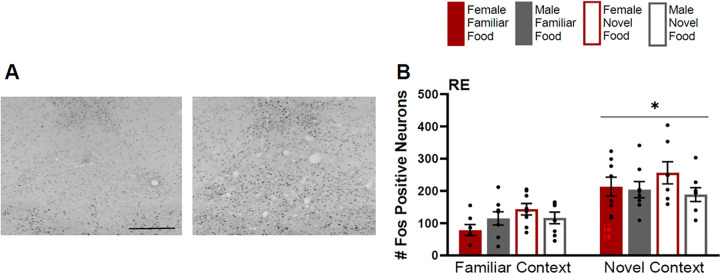
Fos induction in the nucleus reuniens (RE) of the thalamus. **A)** Tissue images stained for Fos of the RE (atlas level 26, midline) for a male tested in a familiar context given a familiar food (left image) and a male tested in a familiar context given a novel food (right image). **B)** Fos induction for each testing condition in the RE. Scale bar in left image=500um. Asterisks indicate p<0.05.

Fos induction in the RE was greater for rats tested in a novel context than rats tested in a familiar context (Figure 7) (F(1,50)=35.977, p<0.01), but there were no differences based on food type (F(1,50)=2.013, p=0.17), or main effect of sex (F(1,50)=1.213, p=0.28). In the novel context, females had slightly higher Fos induction than males, and females that were given novel food had higher Fos induction than males given novel food. However, there were only trends towards significance for interactions of context by sex (F(1,50)=3.327, p=0.074) and sex by food type (F(1,50)=3.127, p=0.084). There were no other interaction effects (context by food type F(1,50)=1.475, p=0.23; context by sex by food type F(1,50)=0.573, p=0.453).

#### Nucleus Accumbens

**Figure 8:**
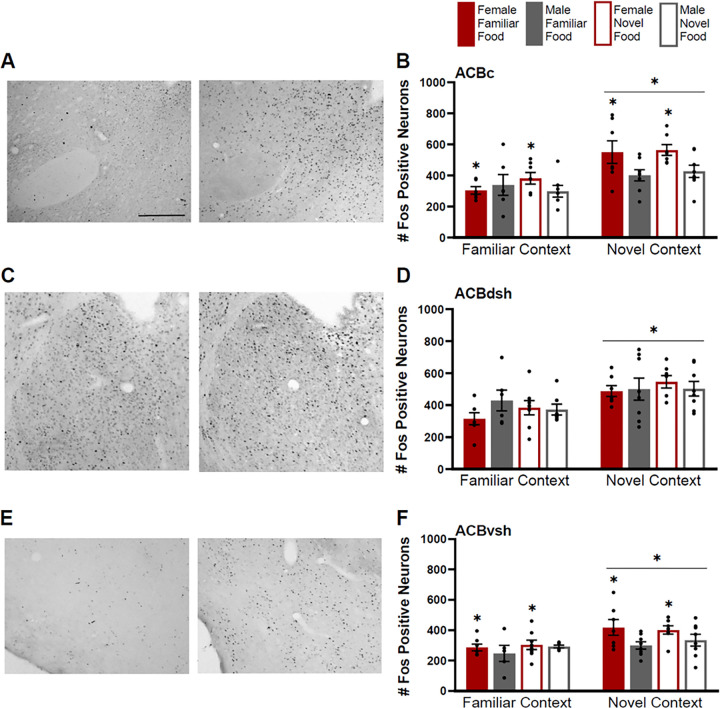
Fos induction in the core (ACBc), dorsal shell (ACBdsh), and ventral shell (ACBvsh) of the nucleus accumbens. **A)** Tissue images stained for Fos of the ACBc (atlas level 14, left side) for a male tested in a novel context given a novel food (left image) and a female tested in a novel context given a novel food (right image). **B)** Fos induction for each testing condition in the ACBc. **C)** Tissue images stained for Fos of the ACBdsh (atlas level 14, right side) for a male tested in a familiar context given a novel food (left image) and a male tested in a novel context given a novel food (right image). **D)** Fos induction for each testing condition in the ACBdsh. **E)** Tissue images stained for Fos of the ACBvsh (atlas level 14, right side) for a male tested in a familiar context given a familiar food (left image) and a female tested in a novel context given a familiar food (right image**). F)** Fos induction for each testing condition in the ACBvsh. Scale bar in upper left image=500um. Asterisks indicate p<0.05.

Fos induction was greater for animals tested in a novel context than for animals tested in a familiar context in all three subregions of the ACB (Figure 8 A–C) (ACBc F(1,47)=22.582, p<0.01; ACBdsh F(1,47)=16.693, p<0.01; ACBvsh F(1,47)=14.67, p<0.01). Additionally, Fos induction was greater for females than males in both the ACBc (F(1,47)=6.829, p=0.012) and ACBvsh (F(1,47)=6.773, p=0.012) (Figure 8 A & C). These sex differences were more obvious in the novel context, however, there was only a trend for interaction of sex and context within the ACBvsh (F1,47)=3.264, p=0.077), but not ACBc (F(1,47)=2.386, p=0.128).

Fos induction in the ACBdsh was similar for both sexes (F(1,47)=0.665, p=−.419). There were no differences in Fos induction based on food type for any subregion of the ACB (ACBc F(1,47)=0.441, p=0.51; ACBdsh F(1,47)=0.125, p=0.725; ACBvsh F(1,47)=0.562, p=0.457), and no significant interactions of factors for any ACB subregion (p>0.05 for all).

#### Medial Prefrontal Cortex

**Figure 9:**
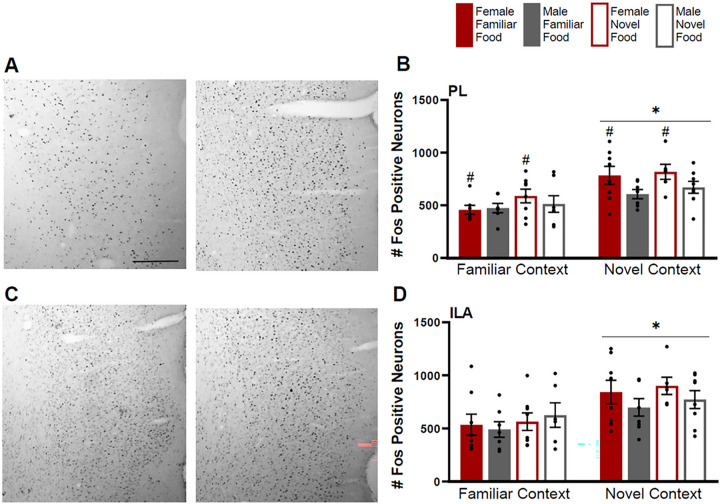
Fos induction in the prelimbic (PL) and infralimbic (ILA) regions of the medial prefrontal cortex. A) Tissue images stained for Fos of the PL (atlas level 8, left side) for a female tested in a familiar context given a familiar food (left image) and female tested in a novel context given a familiar food (right image). B) Fos induction for each testing condition in the PL. C) Tissue images stained for Fos of the ILA (atlas level 9, left side) for a male tested in a familiar context given a familiar food (left image) and male tested in a novel context given a familiar food (right image). D) Fos induction for each testing condition in the ILA. Scale bar in upper left image=500um. Asterisks indicate p<0.05. Pound symbol indicates p=0.06.

Fos induction in the PL was greater for rats tested in a novel context compared to a familiar context (Figure 9 A) (F(1,49)=18.539, p<0.001). In addition, females had slightly higher Fos induction compared to males, however the effect of sex did not reach significance (F(1,49)=3.713, p=0.06). There were main effects of food type (F(1,49)=2.075, p=0.16) or any interactions of factors (context by sex F(1,49)=0.701, p=0.41; context by food type F(1,49)=0.25, p=0.88; sex by food type F(1,49)=0.703, p=0.41; context by sex by food type F(1,49)=0.331, p=0.57).

Fos induction in the ILA was greater for rats tested in a novel context compared to a familiar context (Figure 9 B) (F(1,49)=14.402, p<0.001). There were no differences in Fos induction based on sex (F(1,49)=0.897, p=0.348) or food type (F(1,49)=2.028, p=0.161) and no significant interactions of factors (context by sex F(1,49)=1.958, p=0.17; context by food type F(1,49)=0.019, p=0.89; sex by food type F(1,49)=0.196, p=0.66; context by sex by food type F(1,49)=0.002, p=0.97).

#### Agranular Insular Cortex

**Figure 10:**
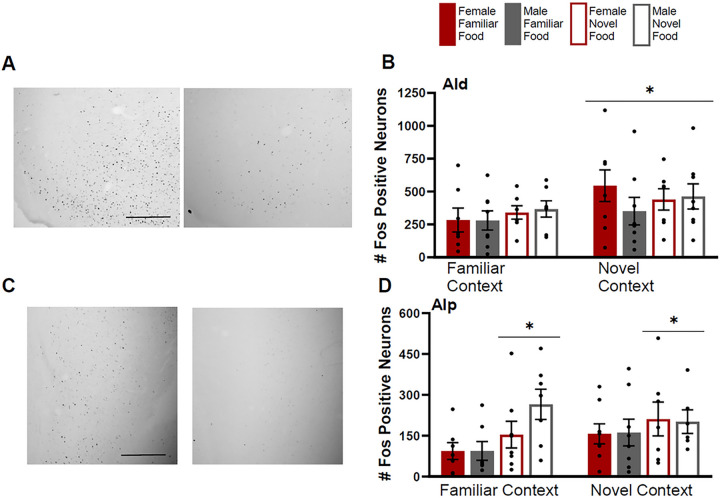
Fos induction in the dorsal agranular insula (AId) and posterior agranular insula (AIp) **A)** Tissue images stained for Fos of the AId (atlas level 10, left side) for a female tested in a familiar context given a familiar food (left image) and a female tested in a novel context given a familiar food (right image). **B)** Fos induction for each testing condition in the AId**. C)** Tissue images stained for Fos of the AIp (atlas level 22, right side) for a male tested in a familiar context given a novel food (left image) and a male tested in a familiar context given a familiar food (right image). **D)** Fos induction for each testing condition in the AIp. Scale bar in upper left image=500um. Asterisks indicate p<0.05.

Fos induction in the AId was greater for rats tested in a novel context compared to rats tested in a familiar context (Figure 10 A) (F(1,49)=4.941, p=0.03). There were no differences in Fos induction based on sex (F(1,49)=0.214, p=0.65) or food type (F(1,49)=0.664, p=0.42) and no significant interactions of factors (context by sex F(1,49)=0.238, p=0.63; context by food type F(1,49)=0.177, p=0.68; sex by food type F(1,49)=1.377, p=0.25; context by sex by food type F(1,49)=0.638, p=0.43)

Fos induction in the AIp was greater for rats given a novel food compared to rats given a familiar food (Figure 10 B) (F(1,49)=6.519, p=0.014). There were no differences in Fos induction based on sex (F(1,49)=0.472, p=0.50) or context (F(1,49)=0.676, p=0.42) and no significant interactions of factors (context by sex F(1,49)=0.577, p=0.45; context by food type F(1,49)=1.302, p=0.26; sex by food type F(1,49)=0.369, p=0.55; context by sex by food type F(1,49)=0.67, p=0.42)

#### Correlations of Fos Induction Between Regions

Bivariate Pearson correlations were conducted within each testing group, to examine the relationship of Fos induction between regions of interest. Females given a familiar food in a familiar context (Table 2; right/above the diagonal) had significant positive and negative correlations. There were positive correlations between CEA subregions, aCEAl with pCEAm, and pCEAm with pCEAl, as well as between CEA and other regions. The BLAa was positively correlated with pCEAl and pCEAc, the BLAp with the aCEAm and the aCEAc, and the LA with the pCEAc. The RE was negatively correlated with the aCEAl. The ACBvsh was positively correlated with the BMAa. The ILA had two significant correlations with other brain regions, a negative correlation with ACBdsh and a positive correlation with the PL. The AId was positively correlated with the PL and ILA. The AIp was negatively correlated with the ACBdsh and positively correlated with the PL, ILA, and AId.

Males given a familiar food in a familiar context (Table 2; left/below the diagonal) had only positive correlations. There were correlations between CEA subregions, pCEAm and aCEAc, pCEAl and aCEAc, as well as pCEAc and aCEAm, aCEAc, pCEAm, and pCEAl. The BMAp was correlated with the BLAa and the LA with the BLAp and BMAp. The PVTp was correlated with the BMAp, the RE with the pCEAm and pCEAc, and ACBdsh with ACBc. The PL was correlated with aCEAm and pCEAc and the ILA with pCEAm, PVTp and RE. The AId was correlated with pCEAl.

Females given a novel food in a familiar context (Table 3; right/above the diagonal) had significant positive and negative correlations. There were positive correlation between pCEAl and aCEAm. In the BMA, there were positive correlations between the anterior and posterior parts and between the BMAp and the pCEAm and BLAp. The RE was positively correlated with the aCEAm. In the ACB, the ACBdsh was negatively correlated with the BLAa and BMAa and the ACBvsh was negatively correlated with the LA. Additionally, the ILA was positively correlated with the RE and PL. The AId was positively correlated with RE and ILA. The AIp was positively correlated with RE, ILA, and AId.

Males given a novel food in a familiar context (Table 3; left/below the diagonal) had significant positive and negative correlations. There were positive correlation between pCEAl and pCEAm. There were positive correlations between the anterior and posterior BLA as well as the BMAa with BLAa and BLAp. The LA was positively correlated with and BLAp, BMAp, and BMAp. The PVTp was negatively correlated with pCEAc. The ACBdsh was positively correlated with ACBc and the ACBvsh was negatively correlated with aCEAl. The PL was positively correlated with RE and the ILA with both PVTp and PL. The AId was positively correlated with aCEAc.

Females given a familiar food in a novel context (Table 4; right/above the diagonal) had significant positive and negative correlations. There were positive correlations between aCEAl and aCEAm, aCEAc and aCEAm, aCEAc and aCEAl, pCEAl and aCEAc, and pCEAc and pCEAl. The anterior and posterior BMA were positively correlated, and the BMAa was correlated with BLAa and the BMAp with BLAa and BLAp. In the PVT there were negative correlations between PVTa and aCEAm and between PVTp and both aCEAl and aCEAc. The ACBc was positively correlated with BLAa, BLAp, and BMAa and the ACBdsh was positively correlated with BLAa and ACBc. There were positive correlations between PL and ACBc and between ILA and ACBc, ACBdsh, and PL. The AId was positively correlated with the ACBc, ACBdsh, PL, and ILA. The AIp was positively correlated with the aCEAm, aCEAc, and RE and negatively correlated with the PVTp.

Males given a familiar food in a novel context (Table 4; left/below diagonal) had only positive correlations. Many CEA subregions were correlated, the aCEAl and aCEAm, aCEAc and aCEAm, aCEAc and aCEAl, pCEAm and aCEAl, pCEAm and aCEAc, pCEAl and aCEAc, pCEAc and aCEAm, pCEAc and aCEAl, pCEAc and aCEAc, and pCEAc and pCEAl. The anterior and posterior BLA were correlated, and the LA was correlated with the BLAa and BMAa. The core and dorsal shell of the ACB were correlated. Additionally, the PL was correlated with pCEAc and the ILA with aCEAm, aCEAc, pCEAl, pCEAc, and PL. The AId was correlated with aCEAm, aCEAc, pCEAc, PVTp, PL, and ILA. The AIp was correlated with the aCEAm, pCEAc, BMAp, PL, and ILA.

Females given a novel food in a novel context (Table 5; right/above the diagonal) had significant positive and negative correlations. There was a negative correlation between anterior and posterior CEAl and CEAl. The anterior and posterior BLA were positively corelated, as well as anterior and posterior BMA. In addition, the BMAa and BMAp were positively correlated with BLAa and BLAp. Also, the LA was positively correlated with BLAa. In the PVT, there was a positive correlation between PVTa and pCEAl, and PVTp and aCEAl, and a negative correlation between PVTp and BMAa. The RE was positively correlated with aCEAl and negatively correlated with pCEAl. The ACBdsh was negatively correlated with BLAp and positively correlated with PVTp, while the ACBvsh was positively correlated with the ACBc. The PL was negatively correlated with aCEAl. Lastly, the AIp was positively correlated with the LA.

Males given a novel food in a novel context (Table 5; left/below the diagonal) had only positive correlations. The CEA subregions had correlations between aCEAl and aCEAm, aCEAc and aCEAm, pCEAm and aCEAc, pCEAl and aCEAm, and pCEAl and aCEAl. The BLAp was correlated with BLAa and BMAa was correlated with BLAa and BLAp. The LA was correlated with both BLAp and BMAa. Additionally, there was a correlation between RE and aCEAm. The ACBdsh was correlated with BLAa and with ACBc and the ACBvsh. The ILA was correlated with aCEAc, ACBvsh, and PL. The AId was correlated with the ACBc. The AIp was correlated with the aCEAm, aCEAc, pCEAl, and pCEAc.

## Discussion

Here, we determined recruitment of several forebrain areas when rats consumed either a novel or familiar food in a novel or familiar context. We analyzed Fos induction in amygdalar, thalamic, striatal, and cortical regions known to be important for appetitive responding, contextual processing, and motivation. Our behavioral preparation was designed to determine separate effects of food and context novelty on both consumption and neuronal activity in each sex. During the food consumption test, similar to previous behavioral findings (Greiner & Petrovich, 2020), both male and female rats ate less of the novel than familiar food, and the groups tested in a novel context ate much less than those tested in a familiar context. Novel context and novel food conditions induced Fos within several regions of interest. Novel context induced Fos robustly in almost every region analyzed, while novel food induced Fos in fewer regions. Some regions analyzed were also differentially recruited in males and females.

## Novel Context

Novel context, as the most salient stimulus, induced robust Fos expression in almost every region analyzed. Rats in the novel context condition had increased Fos induction in all the basolateral complex nuclei of the amygdala (BLAa & BLAp, BMAa & BMAp, LA), the central nucleus of the amygdala (CEAc), all subregions of the ACB (core, vsh, dsh), thalamus (PVTa & RE), medial prefrontal cortex (PL & ILA), and the dorsal AI (AId). Robust Fos expression in all of the basolateral complex nuclei in the novel context condition was expected, given that several of these nuclei are interconnected with the hippocampal formation (HF). The entorhinal cortex, which is important for spatial cognition, and is a component of the trisynaptic circuit, has bidirectional connections with the BMAp, BLAp, and LA (McDonald 1998). Additionally, ventral subiculum (SUBv), which has an established role in contextual encoding (Maren, 1999), has projections to the BMAp and LA (Canteras & Swanson, 1992 Cullinan et al., 1993), which project back (Pikkarainen et al., 1999; Petrovich et al., 2001). The LA, BLA and BMA also receive projections from CA1 (McDonald 1998; Cenquizca & Swanson, 2007), and the LA, BLAp, and BMAp, innervate the CA1 in return (Pikkarainen et al., 1999; Petrovich et al., 2001). The connectivity with the SUBv is of particular interest because it has been previously found to mediate novel stimulus detection—particularly novel environments (Legault & Wise, 2001; Lisman & Grace, 2005). Of note, the BMAa and BLAa, where we observed increased Fos induction in novel context conditions, do not have substantial connections with the HF (Petrovich et al., 2001; Cenquizca & Swanson, 2007), however, the BMAa receives input from the ventromedial PFC (Adhikari et al., 2015) which could relay information from the HF.

Within the CEA, Fos induction in novel context tested groups was specific to the CEAc. This finding is interesting because the CEAc receives substantial inputs from the CA1 (Cenquizca & Swanson, 2007) and the SUBv (Canteras & Swanson, 1992). In addition, contextual information could reach the CEA via multiple relays from the HF (Canteras & Swanson, 1992; Cenquizca & Swanson, 2007), most notably, via inputs from the medial PFC (Hurley et al., 1991; Messanvi et al., 2023) and BLA.

It is important to note that neuronal activity during feeding in a novel environment may be related to processing of novel contextual information, as well as regulation of behavioral responding. Furthermore, some of the amygdala regions could serve as integrators of novel information. The BMAa and CEAc were the only amygdala areas analyzed that responded to both novel context and novel food, which suggests that these regions are processing novelty generally. They could also be controlling feeding in response to novelty rather than responding to specific food or context information. This convergence of novelty processing may be particularly important for driving appropriate behavioral responding. The BMAa sends substantial projections to the CEA (Petrovich et al., 1996) and the CEA is known to both drive (Douglass et al., 2017) and inhibit (Cai et al., 2014) consumption, which is relevant to current findings since consumption levels varied based on novelty condition. The uncertainty of a novel context may also induce responding within safety or defensive circuits. This would align with our findings that novel context exposure recruited both the BLA (both anterior and posterior) and the CEAc. As the BLA R-spondin 2 expressing neurons that inhibit appetitive behavior, and elicit defensive behavior, project to the CEAc (Kim et al. 2016).

Increased neuronal activity for groups tested in a novel context was also robust across thalamic, striatal, and cortical areas analyzed. Within the thalamus, the RE and PVTa had higher Fos induction in the novel context condition. The PVTp also had higher Fos induction in the novel context condition, but the effect was slightly above significance. The recruitment of the RE in a novel context is consistent with its role in contextual memory and novel context encoding. The RE functions as a major thalamic relay for the transfer of information from the medial PFC to the hippocampus (Ferraris et al., 2021; McKenna & Vertes, 2004). The RE is also critical for the formation and retrieval of distinct contextual memories (Ramanathan et al., 2018) and inactivation of RE after fear conditioning resulted in a generalized fear-response to novel contexts (Ramanathan et al., 2018).

The PVTa has been shown previously to control consumption in a novel environment (Cheng et al., 2018). Activation of PVTa neurons that project to the ACB increased consumption in a novel context (Cheng et al., 2018) and activation of PVT GLP-1 receptors, which reduced activity of the PVT to ACB pathway, decreased consumption and food seeking behavior (Ong et al., 2017). It is unclear, however, how this pathway is represented in our findings, where both ACB and PVTa had higher Fos induction in the groups that had suppressed consumption in a novel context. Our methodology was not cell type- or pathway-specific and thus we cannot determine which circuits are represented by the overall activity within the PVTa and ACB.

All subregions of the ACB had increased Fos induction in the novel context condition. The ACB is well positioned to mediate behavioral responding in a novel context. The ACB mediates motivation for reward and is critical for context-mediated appetitive behavior (Raynolds & Berridge 2008). It receives direct HF input (Groenewegen et al., 1999; Canteras et al., 1992) and inactivation of both the ACB core and shell impaired context-induced reinstatement (Fuchs et al., 2008). Additionally, connections to ACB shell from the BLA are required for active avoidance (Ramirez et al., 2015).

The AId had increased Fos induction in groups tested in a novel context. The AId receives direct inputs from the hippocampal field CA1 (Cenquizca & Swanson, 2007) and the basolateral and basomedial nuclei (Kita & Kitai 1990; Petrovich et al., 1996) and sends projections to the basolateral complex nuclei and the central amygdala, particularly strongly to the BLAp and the CEAl (McDonald et al., 1996; Shi & Cassell 1998). Optogenetic inhibition of pathways from the BLA to anterior AI, which includes the AId and ventral AI, enhanced extinction in a conditioned place preference task (Gil-Lievana et al., 2020), implicating this system in reward contextual memory. Additionally, neurons in the right anterior AI are sensitive to aversive stimuli, and activation of these neurons suppresses feeding in mice (Wu et al., 2020). Thus, the AId recruitment in the novel context in the currents study may reflect its role in adaptive inhibition of feeding under uncertainty.

Both medial prefrontal cortical regions analyzed, the PL and ILA, had increased Fos induction in the novel context condition. Both regions receive heavy inputs from the HF (Hoover & Vertes, 2007;Cenquizca & Swanson, 2007; Messanvi et al., 2023) and can impact the HF via the RE (McKenna & Vertes, 2004; Vertes 2002; Hallock et al., 2016). In addition, the mPFC is well positioned to control feeding behavior. One model suggests that local GABAergic inhibition or disinhibition of mPFC glutamatergic projections to the ACBsh and lateral hypothalamus (LHA) controls food consumption (Baldo, 2016). In accordance with this model and prior evidence that PFC mu-opioid stimulation drives feeding through activation of the LHA neurons (Mena et al., 2013), some of the Fos induction within the medial PFC in the current study could represent activity of GABA neurons that are shutting down feeding by inhibiting PFC-LHA pathway. Additionally, given the patterns within the ACBsh in the current study, it is possible that PFC mu-opioid activation of LHA was attenuated by ACB AMPA-receptor activation, which could suppress feeding through inhibition of LHA neurons (Mena et al., 2013; Stratford et al., 1998).

## Novel Food

Novel food, regardless of context, increased Fos induction within the CEA, BMAa, PVTa, and AIp. Novel food induced Fos in fewer regions than novel context. However, the CEA was particularly responsive to food type differences. Novel food recruited all CEA subregions (medial, lateral, and capsular). This matches with previous findings that novel food exposure increases Fos induction in the CEA (Koh et al., 2003). The CEA recruitment may reflect different drives: appetitive drives related to the hunger state of the animal and the palatability of the novel food, or aversive responding related to novel taste avoidance. The CEA has diverse neuronal cell types, which have different roles in the control of feeding, and the methodology used in the current study could not differentiate between them.

Neurons that express protein kinase c-delta (CEA^PKCd^) may be among the Fos-positive cell populations observed within the CEA. The CEA^PKCd^ neurons respond to anorexigenic signals and are required for the inhibition of feeding (Cai et al., 2014). Other neurons in the CEA can promote appetitive behaviors. Activation of the CEA serotonin receptor 2a containing neurons (CEA^HTR2a^) that project to the parabrachial nucleus increased food consumption even in sated rats (Douglass et al., 2017). The CEA^PKCd^ neurons are mostly located within the CEAc and CEAl (Cai et al., 2014; McCullough et al., 2018) and the CEA^HTR2a^ neurons are almost exclusively located within the CEAl (Douglass et al., 2017; Kong & Zweifel, 2021).

Another consideration is that at least some of CEA recruitment may be due to the palatability of the novel food rather than novelty processing or inhibition of eating. The novel food (TestDiet pellets) used in the current study is high in sucrose, and therefore more palatable than the familiar food (Rat Chow). Palatable foods were previously shown to increase Fos induction in the CEA (Park & Carr, 1998, Wu et al., 2014; Parsons et al., 2022) and a subset of CEA neurons that express prepronociceptin (CEA^Pnoc^) mediate palatable food consumption (Hardaway et al., 2019). These CEA^Pnoc^ neurons are located predominantly in the CEAm and CEAl (Hardaway et al., 2019), and their inhibition reduced the latency to feed in a novel environment as well as consumption in home cage after novelty exposure (Hardaway et al., 2019). In the current study, the CEAm and CEAl had selective Fos induction in response to the novel food.

Outside of appetitive responding, stress due to the relative uncertainty of novel food may have recruited populations within the CEAm and CEAl that express corticotropin releasing factor (CRF; also known as CRH) or somatostatin. Neurons that express CRF are important in stress responding (Bale & Vale, 2004) and are largely concentrated within the CEAl, with additional populations within the CEAm and few within the CEAc (Marchant et al., 2007, McCullough et al., 2018). Neurons expressing somatostatin are involved in defensive and fear responses (Yu et al., 2016) and are found in much greater density in the CEAl and CEAm than CEAc (Jolkkonen & Pitkanen, 1998; McCullough et al., 2018). The CEA may be a site where competing drives converge to impact consumption—positive motivation due to food palatability and avoidance due to novelty.

The only other amygdala region analyzed that was selectively recruited by the novel food was the BMAa. The BMAa heavily innervates the CEA (Petrovich et al., 1996), and the two regions had similar patterns in the current study. Previous work found that damage to this region increased latency to approach food in a novel environment (Lukaszewska et al., 1984). Like the CEA, the BMA regulates fear and anxiety responding (Rajbahndari et al., 2021; Amano et al., 2011; de Andrade et al., 2012), including physiological stress responses to social novelty (Mesquita et al., 2016). Additionally, BMA neurons that receive input from the ventromedial PFC are associated with suppression of both freezing and anxiety-state behaviors (Adhikari et al., 2015). Therefore, the BMAa recruitment in the current paradigm may be related to an attempt to override neophobic responding to satisfy physiological needs, given that our animals were food-deprived at the start of testing.

The AIp, like the CEAm and CEAl, had selective activation to a novel food only. The AIp has projects to the CEA (McDonald 1998; Shi & Cassell 1998), and stimulation of the AIp-CEA pathway induced avoidance behavior and suppressed appetitive responding (Gehrlach et al., 2019). Additional AIp projections to the ACB, were also shown to inhibit consumption particularly following internal state changes (Gehrlach et al., 2019).

The only other region that had a greater response to novel food was the PVTa. Like the BMAa and the CEAc, the PVTa was recruited by both novel food and novel context. The patterns of activation within these regions suggests that they are a network that responds to novelty, regardless of whether it is food or context. The PVTa is distinguished by higher expression of galanin (*Gal*) (Gao et al., 2020). *Gal*-positive neurons respond to increased arousal states, and their connections to the ILA are implicated in physiological responses to increased arousal (Gao et al., 2020). Therefore, neurons within the PVTa may have been recruited due to the arousal induced by novelty. Interestingly, the PVTa and PVTp differed in their activation patterns in the current study. There were no group differences within the PVTp due to food type. However, the PVTp had higher Fos induction in the novel context condition, which was close to significance.

## Sex Differences

There were sex differences in Fos induction in the PVTa, the core and ventral shell of the ACB, the CEAc, and the posterior part of the CEAl. Additionally, a difference between sexes in PL activation was close to significance. Sex differences in Fos induction were unexpected given that males and females did not differ behaviorally during the test. This suggests that different neural substrates underlie the same behavior in males and females. It is also possible that the neural activation differences may be predictive of future behavioral sex differences, as males and females differ during habituation to novel contexts (Greiner & Petrovich, 2020).

Females had overall greater Fos induction in the ACBc and ACBvsh. Sex-specific role of the ACB in the control of food consumption has been observed before. Projections to the ACB from a sub-population of LHA neurons that produce melanin-concentrating hormone promoted food consumption for males but not females (Terrill et al., 2020).

Within the PVTa, females given a novel food, regardless of context, had greater Fos expression than their male counterparts. There is prior evidence that stress induced activity of the PVTa differs in females. Ovariectomized females without estradiol replacement had higher stress-induced Fos expression in the PVTa compared to those with replacement (Uneyama et al., 2006). There were key differences between that study and the current that do not allow for direct comparison; the stressor used in the prior study was restraint, while we used novelty and intact females. Nevertheless, our findings contribute to the evidence of sex-specific responding of PVTa neurons.

In the current study, there was a close to significant sex difference in the PL, where females had higher Fos induction than males. Sex differences in medial PFC recruitment has been identified in two related tasks. Higher Fos induction was found in the PL and ILA during context induced renewal of responding to food cues after extinction, though exclusively in males (Anderson & Petrovich, 2017). Another study identified female-specific recruitment of the medial PFC during fear induced hypophagia (Reppucci & Petrovich, 2018). However, the differences in behavioral paradigms between these and the current study preclude further comparisons.

Within the CEA, there were sex differences in two subregions. Fos induction in males was overall greater in the CEAc compared to females regardless of food type or testing context, and in the posterior CEAl, the males given a novel food in a familiar context had greater Fos induction than all other groups. These regions receive distinct inputs from the PB, and the PB-CEAl pathway is implicated in visceral information and the PB-CEAc in nociception (Bernard et al., 1993; Bernard & Besson 1990). Therefore, visceral sensory processing related to hunger sensation or eating during our task, could be the reason why males and females recruited CEAc and CEAl in unique ways.

Similarly, the overall sex differences in the CEAc, ACBc, and ACBvsh in the current study may be related to unique responses to hunger-state. All subjects in our preparation were acutely food deprived before testing. Male and female rodents differ in their physiological responses to food restriction (Kane et al., 2018). Sex dependent neural differences in Fos induction were previously found in the CEAm based on deprivation state (Parsons et al., 2022), and food deprivation recruits the ACB in both sexes. (Parsons et al., 2022; Carr, 2011).

## Network Activation Patterns

The analyses of correlations in Fos induction patterns between our regions of interest, found distinct patterns within each group as well as common patterns across groups. Overall, the CEA was the most correlated with other regions and across its subregions, and this was most apparent in rats that were given a familiar food. The CEA subregions were inter-correlated much less in groups given a novel food compared to groups given a familiar food in the same context. These patterns suggest that in the presence of novel food some inputs to the CEA may suppress local connections and activation patterns in distinct subregions. Considering the complexity of interconnections between the CEA subregions (Jolkkonen & Pitkanen, 1998), it is possible that novel food triggers distinct patterns of local inhibition and disinhibition that cannot be detected by the linear correlational analysis here.

Groups given a novel food had an additional similarity in their correlation patterns. Every group given a novel food, regardless of testing context or sex, had a significant positive correlation between BLAa and BMAa. Interestingly, while BMAa was a region with increased Fos induction to a novel food, BLAa was not. Additionally, the BMAa only sends very light projections to the BLAa (Petrovich et al., 1996) and the two areas are considered to be parts of distinct circuits within the basolateral complex (Swanson & Petrovich, 1998). Therefore, the correlation observed is not due to direct communication between the two regions, but more likely due to parallel functioning systems.

Another overlap between groups occurred in males. All males, regardless of testing conditions, had a positive correlation between core and dorsal shell of the ACB. However, this correlation was not exclusive to males. The ACBc and ACBdsh were also correlated in females tested in a novel context and given a familiar food. Females in the novel/novel condition, instead had a positive correlation between the core and ventral shell. There were no other correlations between ACB subregions in females. These sex differences are interesting because the core and shell have been shown to play opposing roles in a conditioned place preference task with contextual and discrete cues (Ito & Hayen, 2011). Given that our paradigm uses both contextual (novel context) and non-contextual (novel food) cues in tandem, it is possible that, coordination between the ACBc and dorsal shell in males but ventral shell in females is necessary in order to drive appropriate appetitive responses. The inputs from core to shell are heavier than from shell to core (van Dongen et al., 2005) and their balance may be important during habituation to novel foods in novel context.

Another unique pattern in females in the novel/novel condition, was that the PL and ILA were not correlated. The PL and ILA are bidirectionally interconnected, however the connection from PL to ILA is denser than in the other direction (Marek et al., 2018). Activation of PL to ILA pathways enhances fear extinction (Marek et al., 2018), and fear habituation and extinction circuits have been shown to partially overlap, at least in males (Furlong et al., 2016). Thus, it is possible that similar neural mechanisms may be recruited during habituation to a potentially dangerous novel stimulus. The lack of correlation between the PL and ILA may indicate enhanced fear/anxiety and poorer habituation to novelty over time, as previously observed in females (Greiner & Petrovich, 2020).

Females also had more of the negative correlations, with the greatest number in the novel/novel condition. Most negative correlations for females in the novel/novel condition included the CEAl. The anterior and posterior CEAl were negatively correlated with each other and with the PL and RE. The PL and RE are interconnected and are involved in a circuit relaying contextual information from the hippocampus (McKenna & Vertes, 2004; Vertes 2002), but only PL sends direct pathways to the CEA (Vertes, 2004). Negative correlations between CEAl and PL and RE suggest unique contextual processing in the female novel/novel condition.

Both female groups given a familiar food had negative correlations between anterior CEA subregions and one of the midline thalamic nuclei analyzed. The familiar/familiar group had a negative correlation between anterior CEAl and RE, while the familiar food/novel context group had negative correlations between anterior CEAm and PVTa and between both anterior CEAl and anterior CEAc and PVTp. Since there are no direct anatomical connections between the RE and CEA, the activation of one region is not directly silencing the other; however, it is possible that a shared input impacts these regions in opposite ways.

The females in the familiar/familiar condition also had unique negative correlations between the ACBdsh and ILA and between the ACBdsh and AIp. Sex specific responding has been found in the ILA to ACB shell pathway. Stimulation of this pathway suppressed conditioned taste aversion in males only, but increased sucrose preference for both sexes (Hurley & Carelli, 2020). The AIp sends some projections to the ACB shell (Reynolds & Zahm, 2005), and a negative correlation between these regions is expected, given that the ACBsh drives motivation to consume foods (Castro et al., 2016) while AIp-ACB stimulation suppresses food consumption (Gehrlach et al., 2019).

Negative ACB shell correlations were present in females in the novel food/familiar context condition as well, though they were exclusively with the amygdala basolateral complex nuclei (ACBdsh and BLAa, ACBdsh and BMAa, ACBvsh and LA). Activation of the BLA-ACB pathway is thought to facilitate reward learning (Dieterich et al., 2021). An inverse relationship in activity between these two regions could suggest that BLA may be active in other circuits, potentially driving aversive responding to the novel food, rather than stimulating reward responding through the ACB.

Females in the familiar food/novel context group had a negative correlation between Fos induction in the AIp and PVTp. The AIp sends dense projections to the PVTp (Li & Kirouac, 2012) and is thought to communicate gustatory and viscerosensory information (Kirouac, 2015). The negative relationship between these areas may be indicate suppression of response to physiological hunger for females in a novel context.

Another unique pattern in groups with no novel stimuli, was no overlap in region correlations between females and males in the familiar/familiar groups. That suggests that males and females have distinct processing for food consumption at baseline.

## Proposed Functional Circuitries

**Figure 11:**
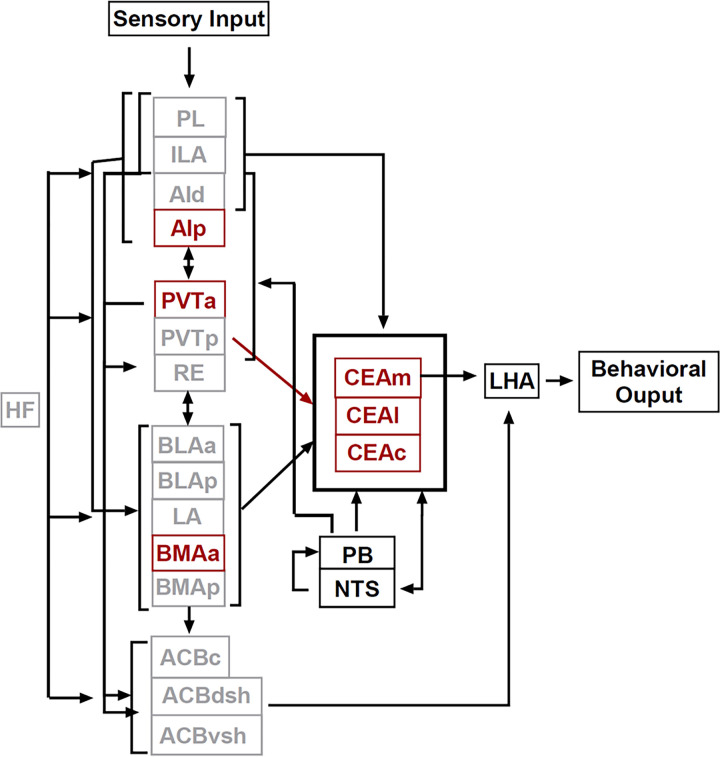
The diagram shows the proposed circuitry for feeding inhibition in response to a novel food. Areas that were activated in response to a novel food during the consumption test are shown in red. Areas that were not activated are in gray. For clarity some connections not shown. Within this circuitry, females had greater activation than males in PVTa and males had greater activation than females in CEAc.

**Figure 12:**
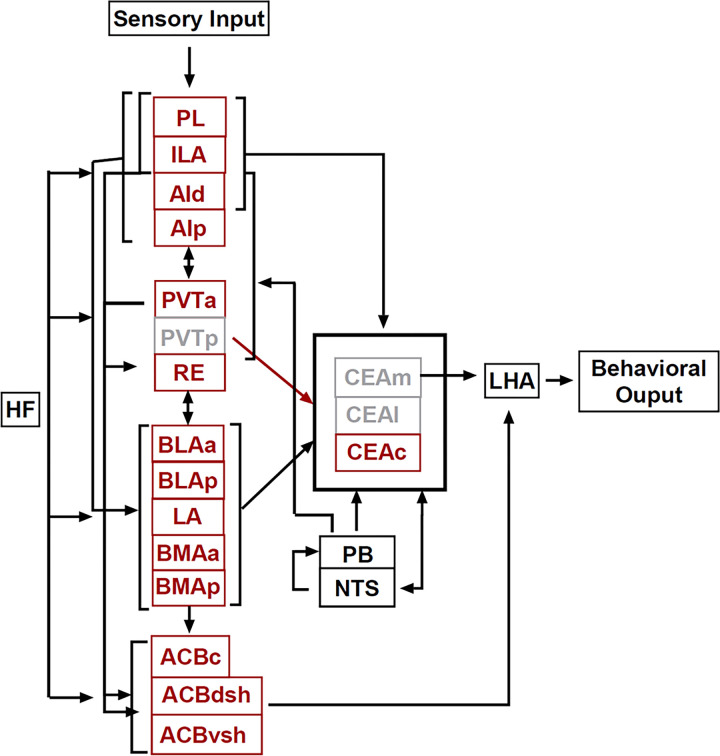
The diagram shows the proposed circuitry for feeding inhibition in a novel context. Areas that were activated in a novel context are shown in red. Areas that were not activated are in gray. For clarity some connections are not shown. Within this circuitry, females had greater activation than males in ACBc & ACBvsh and males had greater activation than females in CEAc.

Based on the activation patterns found in the current study and known connectivity, we have identified two distinct, functional circuitries within a larger network that controls feeding behavior. The proposed circuitry for control of feeding when consuming a novel food (hereby referred to as the *novel food circuitry*) is shown in Fig. 11. The proposed circuitry for control of feeding in a novel context (hereby referred to as the *novel context circuitry*) is shown in Fig. 12. Within each circuitry we identified regions that were activated differently in males and females.

Each circuitry includes a subset of areas identified that were selectively activated during novel context or novel food exposure and are known to transmit specific information about context and feeding. We additionally included regions that were not analyzed in the current study but are anatomically connected within the proposed circuitries: the HF, which is critical for contextual processing and encoding, the nucleus of the solitary tract (NTS) and parabrachial nucleus (PB), which are necessary for transmitting gustatory, taste, and visceral sensory information, and the lateral hypothalamus (LHA) which is considered a motivation-cognition interface in the control of feeding behavior (for review see Petrovich, 2018).

These two distinct circuitries have key regions of overlap—the PVT, CEA, & BMAa—and we postulate that when both circuitries are active, they have a cumulative impact on the inhibition of feeding. Patterns of activity within the PVT, CEA, and BMAa suggest that they are drivers of eating control, regardless of whether feeding inhibition is due to novel taste or novel context. We speculate that the *novel food circuitry* is a subset of the *novel context circuitry*, and that the main difference is heavy mediation by cortical inputs in the *novel context circuit*. The CEA is a place of convergence of competing drives: hunger and hedonic information that would increase feeding, and stress, anxiety and arousal information that would suppress feeding (Petrovich, 2018). The proposed model suggests that in the *novel food circuitry*, the CEA drives feeding inhibition, whereas in the *novel context circuit* both CEA and ACB drive feeding inhibition. Therefore, when animals consume a novel food in a novel context, both circuitries would be engaged, and both the ACB and CEA would mediate feeding inhibition.

## Implications

The current study identified distinct circuits that underlie food and context novelty processing during consumption. The identified neural circuitries mediate the control of competing motivations and behaviors: appetitive drive to feed and acquire reward versus avoidance of uncertainty. Additionally, we found sex-specific activation patterns that may be predictive of enhanced hypophagia in females during habituation to eating in an uncertain, novel environment. These findings have important implications for future functional studies and our better understanding of neural and behavioral mechanisms underlying maladaptive eating behaviors and psychopathology in each sex.

## Figures and Tables

**Table 1:** The rostro-caudal extent of each brain region analyzed. Atlas levels refer to the Swanson rat brain atlas (2018).

Brain Region	Analyzed Subregions	Representative Atlas Level(s)	Distance from Bregma
**CEA**	*CEAm*	25, 26, 27, 28	−1.53, −1.78, −2, −2.45
	*CEAc*		
	*CEAl*	26, 27, 28	−1.78, −2, −2.45
**BLA**	*BLAa*	27	−2
	*BLAp*	30	−3.25
**BMA**	*BMAa*	26	−1.78
	*BMAp*	30	−3.25
**LA**	-	30	−3.25
**PVT**	*PVTa*	26	−1.78
	*PVTp*	31	−3.7
**RE**	-	26	−1.78
**ACB**	*ACBc*	13	+ 1.2
	*ACBdsh*		
	*ACBvsh*		
**mPFC**	*PL*	8	+ 3.2
	*ILA*	9	+ 2.8
**AI**	*AId*	10	+ 2.15
	*AIp*	22	−0.83

## Data Availability

The data that support the findings of this study are available from the corresponding author upon reasonable request.

## References

[1] Adhikari A., Lerner T. N., Finkelstein J., Pak S., Jennings J. H., Davidson T. J., Ferenczi E., Gunaydin L. A., Mirzabekov J. J., Ye L., Kim S.-Y., Lei A., & Deisseroth K. (2015). Basomedial amygdala mediates top-down control of anxiety and fear. Nature, 527(7577), 179–185. 10.1038/nature15698 26536109 PMC4780260

[2] Allen GV, Saper CB, Hurley KM, Cechetto DF (1991). Organization of visceral and limbic connections in the insular cortex of the rat. J Comp Neurol 311: 1–16. 1719041 10.1002/cne.903110102

[3] Anderson L. C., & Petrovich G. D. (2017). Sex specific recruitment of a medial prefrontal cortex-hippocampal-thalamic system during context-dependent renewal of responding to food cues in rats. Neurobiology of Learning and Memory, 139, 11–21. 27940080 10.1016/j.nlm.2016.12.004 PMC5334368

[4] Baldo BA. Prefrontal Cortical Opioids and Dysregulated Motivation: A Network Hypothesis. Trends Neurosci. 2016;39(6):366–377. doi:10.1016/j.tins.2016.03.004 27233653 PMC5818385

[5] Bale T. L., & Vale W. W. (2004). CRF and CRF receptors: role in stress responsivity and other behaviors. Annual review of pharmacology and toxicology, 44, 525–557. 10.1146/annurev.pharmtox.44.101802.121410 14744257

[6] Bechara A., & Damasio A. R. (2005). The somatic marker hypothesis: A neural theory of economic decision. Games and Economic Behavior, 52(2), 336–372.

[7] Bermudez-Rattoni F. (2014). The forgotten insular cortex: its role on recognition memory formation. Neurobiology of learning and memory, 109, 207–216 24406466 10.1016/j.nlm.2014.01.001

[8] Bernard J-F, Alden M, Besson J-M (1993) The organization of the efferent projections from the pontine parabrachial area to the amygdaloid complex: A Phaseolus vulgaris leucoagglutinin (PHA-L) study in the rat. J Comp Neurol, 329: 201–229. 8454730 10.1002/cne.903290205

[9] Bernard J. F., & Besson J. M. (1990). The spino(trigemino)pontoamygdaloid pathway: electrophysiological evidence for an involvement in pain processes. Journal of neurophysiology, 63(3), 473–490. 10.1152/jn.1990.63.3.473 2329357

[10] Bhatnagar S. & Dallman M. F. (1999). The paraventricular nucleus of the thalamus alters rhythms in core temperature and energy balance in a state-dependent manner. Brain Research, 851(1–2), 66–75. 10642829 10.1016/s0006-8993(99)02108-3

[11] Bozarth M. A., & Wise R. A. (1981). Intracranial self-administration of morphine into the ventral tegmental area in rats. Life sciences, 28(5), 551–555. 10.1016/0024-3205(81)90148-x 7207031

[12] Brog J. S., Salyapongse A., Deutch A. Y., & Zahm D. S. (1993). The patterns of afferent innervation of the core and shell in the “accumbens” part of the rat ventral striatum: immunohistochemical detection of retrogradely transported fluoro-gold. Journal of comparative neurology, 338(2), 255–278. 8308171 10.1002/cne.903380209

[13] Cai H, Haubensak W, Anthony TE, Anderson DJ (2014). Central amygdala PKC-δ(+) neurons mediate the influence of multiple anorexigenic signals. Nature Neuroscience., 17, 9, 1240–8. doi: 10.1038/nn.3767. 25064852 PMC4146747

[14] Canteras N. S., & Swanson L. W. (1992). Projections of the ventral subiculum to the amygdala, septum, and hypothalamus: A PHAL anterograde tract-tracing study in the rat. The Journal of Comparative Neurology, 324(2), 180–194. 1430328 10.1002/cne.903240204

[15] Carr K. D. (2011). Food scarcity, neuroadaptations, and the pathogenic potential of dieting in an unnatural ecology: binge eating and drug abuse. Physiology & behavior, 104(1), 162–167. 10.1016/j.physbeh.2011.04.023 21530562 PMC3107914

[16] Castro DC, Berridge KC. Opioid hedonic hotspot in nucleus accumbens shell: mu, delta, and kappa maps for enhancement of sweetness “liking” and “wanting”. J Neurosci. 2014 Mar 19;34(12):4239–50. doi: 10.1523/JNEUROSCI.4458-13.2014. 24647944 PMC3960467

[17] Cenquizca L. A., & Swanson L. W. (2007). Spatial organization of direct hippocampal field CA1 axonal projections to the rest of the cerebral cortex. Brain Research Reviews, 56(1), 1–26. 17559940 10.1016/j.brainresrev.2007.05.002 PMC2171036

[18] Cheng J., Wang J., Ma X., Ullah R., Shen Y., & Zhou Y. (2018). Anterior Paraventricular Thalamus to Nucleus Accumbens Projection Is Involved in Feeding Behavior in a Novel Environment. Frontiers in Molecular Neuroscience, 11. 29930498 10.3389/fnmol.2018.00202 PMC5999750

[19] Christie M. J., Summers R. J., Stephenson J. A., Cook C. J., & Beart P. M. (1987). Excitatory amino acid projections to the nucleus accumbens septi in the rat: a retrograde transport study utilizingd [3H] aspartate and [3H] GABA. Neuroscience, 22(2), 425–439. 2823173 10.1016/0306-4522(87)90345-9

[20] Cole S., Powell D. J., & Petrovich G. D. (2013). Differential recruitment of distinct amygdalar nuclei across appetitive associative learning. Learning & Memory, 20(6), 295–299. 23676201 10.1101/lm.031070.113 PMC3677082

[21] Cole S., Keefer S. E., Anderson L. C., & Petrovich G. D. (2020). Medial Prefrontal Cortex Neural Plasticity, Orexin Receptor 1 Signaling, and Connectivity with the Lateral Hypothalamus Are Necessary in Cue-Potentiated Feeding. J Neurosci, 40(8), 1744–1755. 10.1523/JNEUROSCI.1803-19.2020 31953368 PMC7046338

[22] Cullinan W. E., Herman J. P., & Watson S. J. (1993). Ventral subicular interaction with the hypothalamic paraventricular nucleus: Evidence for a relay in the bed nucleus of the Stria terminalis. The Journal of Comparative Neurology, 332(1), 1–20. 10.1002/cne.903320102 7685778

[23] de Andrade J. S., Abrão R. O., Céspedes I. C., Garcia M. C., Nascimento J. O. G., Spadari-Bratfisch R. C., Melo L. L., da Silva R. C. B., & Viana M. B. (2012). Acute restraint differently alters defensive responses and fos immunoreactivity in the rat brain. Behavioural Brain Research, 232(1), 20–29. 10.1016/j.bbr.2012.03.034 22487246

[24] Dieterich A., Floeder J., Stech K., Lee J., Srivastava P., Barker D. J., & Samuels B. A. (2021). Activation of basolateral amygdala to nucleus accumbens projection neurons attenuates chronic corticosterone-induced behavioral deficits in male mice. Frontiers in behavioral neuroscience, 17. 10.3389/fnbeh.2021.643272 PMC7943928 33716685

[25] Dong X., Li S., & Kirouac G. J. (2017). Collateralization of projections from the paraventricular nucleus of the thalamus to the nucleus accumbens, bed nucleus of the stria terminalis, and central nucleus of the amygdala. Brain structure & function, 222(9), 3927–3943. 10.1007/s00429-017-1445-8 28528379

[26] Douglass A. M., Kucukdereli H., Ponserre M., Markovic M., Gründemann J., Strobel C., . . . Klein R. (2017). Central amygdala circuits modulate food consumption through a positive-valence mechanism. 10.1038/nn.4623 28825719

[27] Ferraris M., Cassel J. C., Pereira de Vasconcelos A., Stephan A., & Quilichini P. P. (2021). The nucleus reuniens, a thalamic relay for cortico-hippocampal interaction in recent and remote memory consolidation. Neuroscience and biobehavioral reviews, 125, 339–354. 10.1016/j.neubiorev.2021.02.025 33631314

[28] Fuchs R. A., Ramirez D. R., & Bell G. H. (2008). Nucleus accumbens shell and core involvement in drug context-induced reinstatement of cocaine seeking in rats. Psychopharmacology, 200(4), 545–556. 10.1007/s00213-008-1234-4 18597075 PMC2613506

[29] Gabbott P. L., Warner T. A., Jays P. R., Salway P., & Busby S. J. (2005). Prefrontal cortex in the rat: Projections to subcortical autonomic, motor, and limbic centers. The Journal of Comparative Neurology, 492(2), 145–177. 16196030 10.1002/cne.20738

[30] Gao C., Leng Y., Ma J., Rooke V., Rodriguez-Gonzalez S., Ramakrishnan C., Deisseroth K., & Penzo M. A. (2020). Two genetically, anatomically and functionally distinct cell types segregate across anteroposterior axis of paraventricular thalamus. Nature Neuroscience, 23(2), 217–228. 10.1038/s41593-019-0572-3 31932767 PMC7007348

[31] Gehrlach D. A., Dolensek N., Klein A. S., Roy Chowdhury R., Matthys A., Junghänel M., ... & Gogolla, N. (2019). Aversive state processing in the posterior insular cortex. Nature neuroscience, 22(9), 1424–1437. 31455886 10.1038/s41593-019-0469-1

[32] Gil-Lievana E., Balderas I., Moreno-Castilla P., Luis-Islas J., McDevitt R. A., Tecuapetla F., Gutierrez R., Bonci A., & Bermúdez-Rattoni F. (2020). Glutamatergic basolateral amygdala to anterior insular cortex circuitry maintains rewarding contextual memory. Communications Biology, 3(1), 139. 10.1038/s42003-020-0862-z 32198461 PMC7083952

[33] Gogolla N. (2017). The insular cortex. Curr Biol, 27(12), R580–R586. 10.1016/j.cub.2017.05.010 28633023

[34] Greiner E. M., & Petrovich G. D. (2020). The effects of novelty on food consumption in male and female rats. Physiology & Behavior, 223, 112970. doi:10.1016/j.physbeh.2020.112970 32464137 PMC7358116

[35] Groenewegen H. J., Wright C. I., Beijer A. V., & Voorn P. (1999). Convergence and segregation of ventral striatal inputs and outputs. Annals of the New York Academy of Sciences, 877, 49–63. 10.1111/j.1749-6632.1999.tb09260.x 10415642

[36] Hallock H. L., Wang A., & Griffin A. L. (2016). Ventral Midline Thalamus Is Critical for Hippocampal-Prefrontal Synchrony and Spatial Working Memory. The Journal of neuroscience: the official journal of the Society for Neuroscience, 36(32), 8372–8389. 10.1523/JNEUROSCI.0991-16.2016 27511010 PMC4978800

[37] Hardaway JA, Halladay LR, Mazzone CM, Pati D, Bloodgood DW, Kim M, Jensen J, DiBerto JF, Boyt KM, Shiddapur A, Erfani A, Hon OJ, Neira S, Stanhope CM, Sugam JA, Saddoris MP, Tipton G, McElligott Z, Jhou TC, Stuber GD, Bruchas MR, Bulik CM, Holmes A, Kash TL. Central Amygdala Prepronociceptin-Expressing Neurons Mediate Palatable Food Consumption and Reward. Neuron. 2019 Jun 5;102(5):1037–1052.e7. doi: 10.1016/j.neuron.2019.03.037 31029403 PMC6750705

[38] Hintiryan H., Bowman I., Johnson D. L., Korobkova L., Zhu M., Khanjani N., … & Dong H. W. (2021). Connectivity characterization of the mouse basolateral amygdalar complex. Nature communications, 12(1), 2859. 10.1038/s41467-021-22915-5 PMC8129205 34001873

[39] Hoover W. B., & Vertes R. P. (2007). Anatomical analysis of afferent projections to the medial prefrontal cortex in the rat. Brain structure & function, 212(2), 149–179. 10.1007/s00429-007-0150-4 17717690

[40] Hurley S. W., & Carelli R. M. (2020). Activation of Infralimbic to Nucleus Accumbens Shell Pathway Suppresses Conditioned Aversion in Male But Not Female Rats. The Journal of neuroscience : the official journal of the Society for Neuroscience, 40(36), 6888–6895. 10.1523/JNEUROSCI.0137-20.2020 32727819 PMC7470915

[41] Hurley K. M., Herbert H., Moga M. M., & Saper C. B. (1991). Efferent projections of the infralimbic cortex of the rat. The Journal of comparative neurology, 308(2), 249–276. 10.1002/cne.903080210 1716270

[42] Ito R., & Hayen A. (2011). Opposing roles of nucleus accumbens core and shell dopamine in the modulation of limbic information processing. J Neurosci, 31(16), 6001–6007. 10.1523/JNEUROSCI.6588-10.2011 21508225 PMC3160482

[43] Jolkkonen E., & Pitkanen A. (1998). Intrinsic connections of the rat amygdaloid complex: Projections originating in the central nucleus. The Journal of Comparative Neurology, 395(1), 53–72. doi:10.1002/(sici)1096-9861(19980525)395:13.0.co;2-g 9590546

[44] Kane A. E., Sinclair D. A., Mitchell J. R., & Mitchell S. J. (2018). Sex differences in the response to dietary restriction in rodents. Current opinion in physiology, 6, 28–34. 10.1016/j.cophys.2018.03.008 31231711 PMC6588196

[45] Kelley A. E. (2004). Ventral striatal control of appetitive motivation: role in ingestive behavior and reward-related learning. Neuroscience and biobehavioral reviews, 27(8), 765–776. 10.1016/j.neubiorev.2003.11.015 15019426

[46] Kim J, Pignatelli M, Xu S, Itohara S, Tonegawa S. (2016). Antagonistic negative and positive neurons of the basolateral amygdala. Nature Neuroscience, 19, 12, 1636–1646. doi: 10.1038/nn.4414. 27749826 PMC5493320

[47] Kirouac G. J. (2015). Placing the paraventricular nucleus of the thalamus within the brain circuits that control behavior. Neuroscience & Biobehavioral Reviews, 56, 315–329. 10.1016/j.neubiorev.2015.08.005 26255593

[48] Kita H., & Kitai S. T. (1990). Amygdaloid projections to the frontal cortex and the striatum in the rat. The Journal of comparative neurology, 298(1), 40–49. 10.1002/cne.902980104 1698828

[49] Koh M. T., Wilkins E. E., & Bernstein I. L. (2003). Novel Tastes Elevate c-fos Expression in the Central Amygdala and Insular Cortex: Implication for Taste Aversion Learning. Behavioral Neuroscience, 117(6), 1416–1422. 14674859 10.1037/0735-7044.117.6.1416

[50] Kong M. S., & Zweifel L. S. (2021). Central amygdala circuits in valence and salience processing. Behavioural brain research, 410, 113355. 10.1016/j.bbr.2021.113355 33989728 PMC8178205

[51] Krettek J. E., & Price J. L. (1977). Projections from the amygdaloid complex and adjacent olfactory structures to the entorhinal cortex and to the subiculum in the rat and cat. The Journal of Comparative Neurology, 172(4), 723–752. 10.1002/cne.901720409 838896

[52] Land B. B., Narayanan N. S., Liu R.-J., Gianessi C. A., Brayton C. E., M Grimaldi D., Sarhan M., Guarnieri D. J., Deisseroth K., Aghajanian G. K., & DiLeone R. J. (2014). Medial prefrontal D1 dopamine neurons control food intake. Nature Neuroscience, 17(2), 248–253. 10.1038/nn.3625 24441680 PMC3968853

[53] Legault M., & Wise R. A. (2001). Novelty-evoked elevations of nucleus accumbens dopamine: Dependence on impulse flow from the ventral subiculum and glutamatergic neurotransmission in the ventral tegmental area. European Journal of Neuroscience, 13(4), 819–828. 10.1046/j.0953-816x.2000.01448.x 11207817

[54] Li S., & Kirouac G. J. (2008). Projections from the paraventricular nucleus of the thalamus to the forebrain, with special emphasis on the extended amygdala. The Journal of Comparative Neurology, 509(1), 136–140. 10.1002/cne.21502 18022956

[55] Li S., & Kirouac G. J. (2012). Sources of inputs to the anterior and posterior aspects of the paraventricular nucleus of the thalamus. Brain Structure and Function, 217, 257–273. 22086160 10.1007/s00429-011-0360-7

[56] Lin J., Roman C., Andre J. S., & Reilly S. (2009). Taste, olfactory and trigeminal neophobia in rats with forebrain lesions. Brain Research, 1251, 195–203. 19059225 10.1016/j.brainres.2008.11.040 PMC2722112

[57] Lin J., Roman C., Arthurs J., & Reilly S. (2012). Taste neophobia and c-Fos expression in the rat brain. Brain Research, 1448, 82–88. 22405689 10.1016/j.brainres.2012.02.013 PMC3313599

[58] Linley S. B., Athanason A. C., Rojas A. K. P., & Vertes R. P. (2021). Role of the reuniens and rhomboid thalamic nuclei in anxiety-like avoidance behavior in the rat. Hippocampus, 31(7), 756–769. 10.1002/hipo.23302 33476077

[59] Lisman J. E., & Grace A. A. (2005). The hippocampal-VTA loop: Controlling the entry of information into long-term memory. Neuron, 46(5), 703–713. 10.1016/j.neuron.2005.05.002 15924857

[60] Lukaszewska I., Korczynski R., Kostarczyk E., & Fonberg E. (1984). Food-motivated behavior in rats with cortico-basomedial amygdala damage. Behavioral Neuroscience, 98(3), 441–451. 10.1037/0735-7044.98.3.441 6732925

[61] Marchant N. J., Densmore V. S., & Osborne P. B. (2007). Coexpression of prodynorphin AND corticotrophin-releasing hormone in the rat central amygdala: evidence of two distinct endogenous Opioid systems in the lateral division. The Journal of Comparative Neurology, 504(6), 702–715. Doi:10.1002/cne.21464 17722034

[62] McCullough KM, Morrison FG, Hartmann J, Carlezon WA Jr, Ressler KJ. Quantified Coexpression Analysis of Central Amygdala Subpopulations. eNeuro. 2018 Feb 6;5(1).0010–18.2018. doi: 10.1523/ENEURO.0010-18.2018.. PMC5810038 29445764

[63] McDonald A. J. (1998). Cortical pathways to the mammalian amygdala. Progress in neurobiology, 55(3), 257–332. 10.1016/s0301-0082(98)00003-3 9643556

[64] McKenna J. T., & Vertes R. P. (2004). Afferent projections to nucleus reuniens of the thalamus. The Journal of comparative neurology, 480(2), 115–142. 10.1002/cne.20342 15514932

[65] Mena J. D., Selleck R. A., & Baldo B. A. (2013). Mu-opioid stimulation in rat prefrontal cortex engages hypothalamic orexin/hypocretin-containing neurons, and reveals dissociable roles of nucleus accumbens and hypothalamus in cortically driven feeding. The Journal of neuroscience : the official journal of the Society for Neuroscience, 33(47), 18540–18552. 10.1523/JNEUROSCI.3323-12.2013 24259576 PMC3834058

[66] Marek R., Xu L., Sullivan R. K. P., & Sah P. (2018). Excitatory connections between the prelimbic and infralimbic medial prefrontal cortex show a role for the prelimbic cortex in fear extinction. Nature neuroscience, 21(5), 654–658. 10.1038/s41593-018-0137-x 29686260

[67] Messanvi K. F., Berkun K., Perkins A., & Chudasama Y. (2023). Parallel Pathways Provide Hippocampal Spatial Information to Prefrontal Cortex. The Journal of neuroscience : the official journal of the Society for Neuroscience, 43(1), 68–81. 10.1523/JNEUROSCI.0846-22.2022 36414405 PMC9838712

[68] Mesquita L. T., Abreu A. R., de Abreu A. R., de Souza A. A., de Noronha S. R., Silva F. C., Campos G. S., Chianca D. A., & de Menezes R. C. (2016). New insights on amygdala: Basomedial amygdala regulates the physiological response to social novelty. Neuroscience, 330, 181–190. 10.1016/j.neuroscience.2016.05.053 27261213

[69] Morales I., & Berridge K. C. (2020). ‘Liking’ and ‘wanting’ in eating and food reward: Brain mechanisms and clinical implications. Physiology & behavior, 227, 113152. 10.1016/j.physbeh.2020.113152 32846152 PMC7655589

[70] Nachman M., & Ashe J. H. (1974). Effects of basolateral amygdala lesions on neophobia, learned taste aversions, and sodium appetite in rats. Journal of Comparative and Physiological Psychology, 87(4), 622–643. 10.1037/h0036973 4426986

[71] Ong Z. Y., Liu J. J., Pang Z. P., & Grill H. J. (2017). Paraventricular Thalamic Control of Food Intake and Reward: Role of Glucagon-Like Peptide-1 Receptor Signaling. Neuropsychopharmacology : official publication of the American College of Neuropsychopharmacology, 42(12), 2387–2397. 10.1038/npp.2017.150 28811669 PMC5645740

[72] Park TH, Carr KD. (1998) Neuroanatomical patterns of fos-like immunoreactivity induced by a palatable meal and meal-paired environment in saline- and naltrexone-treated rats. Brain Res., 14, 805(1–2),169–80. doi: 10.1016/s0006-8993(98)00719-7. 9733960

[73] Parsons W., Greiner E., Buczek L., Migliaccio J., Corbett E., Madden A. M., & Petrovich G. D. (2022). Sex differences in activation of extra-hypothalamic forebrain areas during hedonic eating. Brain structure & function, 227(8), 2857–2878. 36258044 10.1007/s00429-022-02580-0 PMC9724631

[74] Parsons M. P., Li S., & Kirouac G. J. (2007). Functional and anatomical connection between the paraventricular nucleus of the thalamus and dopamine fibers of the nucleus accumbens. The Journal of comparative neurology, 500(6), 1050–1063. 10.1002/cne.21224 17183538

[75] Petrovich G. D. (2018). Feeding behavior survival circuit: Anticipation & competition. Current Opinion in Behavioral Sciences, 24, 137–142 31086808 10.1016/j.cobeha.2018.09.007 PMC6510508

[76] Petrovich G. D., Canteras N. S., Swanson L. W. (2001). Combinatorial amygdalar inputs to hippocampal domains and hypothalamic behavior systems. Brain Research Review, 38:247–289. 10.1016/s0165-0173(01)00080-7 11750934

[77] Petrovich G. D., Risold P. Y., & Swanson L. W. (1996). Organization of projections from the basomedial nucleus of the amygdala: A phal study in the rat. The Journal of Comparative Neurology, 374(3), 387–420. 10.1002/(sici)1096-9861(19961021)374:3&lt;387::aid-cne6&gt;3.0.co;2-y 8906507

[78] Petrovich G. D., Ross C. A., Holland P. C., & Gallagher M. (2007). Medial prefrontal cortex is necessary for an appetitive contextual conditioned stimulus to promote eating in sated rats. Journal of Neuroscience, 27(24), 6436–6441. 17567804 10.1523/JNEUROSCI.5001-06.2007 PMC3219438

[79] Petrovich G. D., Ross C. A., Mody P., Holland P. C., & Gallagher M. (2009). Central, But Not Basolateral, Amygdala Is Critical for Control of Feeding by Aversive Learned Cues. Journal of Neuroscience, 29(48), 15205–15212. doi: 10.1523/jneurosci.3656-09.2009 19955373 PMC3321540

[80] Pikkarainen M., Ronkko S., Savander V., Insausti R., & Pitkanen A. (1999). Projections from the lateral, basal, and accessory basal nuclei of the amygdala to the hippocampal formation in rat. The Journal of Comparative Neurology, 403(2), 229–260. 10.1002/(sici)1096-9861(19990111)403:2&lt;229::aid-cne7&gt;3.0.co;2-p 9886046

[81] Pitkänen A., Savander V., & LeDoux J. E. (1997). Organization of intra-amygdaloid circuitries in the rat: An emerging framework for understanding functions of the amygdala. Trends in Neurosciences, 20(11), 517–523. 10.1016/s0166-2236(97)01125-9 9364666

[82] Quirk G. J., Likhtik E., Pelletier J. G., & Paré D. (2003). Stimulation of medial prefrontal cortex decreases the responsiveness of central amygdala output neurons. Journal of Neuroscience, 23(25), 8800–8807. 14507980 10.1523/JNEUROSCI.23-25-08800.2003 PMC6740415

[83] Rajbhandari A. K., Octeau C. J., Gonzalez S., Pennington Z. T., Mohamed F., Trott J., Chavez J., Ngyuen E., Keces N., Hong W. Z., Neve R. L., Waschek J., Khakh B. S., & Fanselow M. S. (2021). A basomedial amygdala to intercalated cells microcircuit expressing PACAP and its receptor PAC1 regulates contextual fear. The Journal of Neuroscience, 41(15), 3446–3461. 10.1523/jneurosci.2564-20.2021 33637560 PMC8051692

[84] Ramanathan K. R., Ressler R. L., Jin J., & Maren S. (2018). Nucleus reuniens is required for encoding and retrieving precise, hippocampal-dependent contextual fear memories in rats. The Journal of Neuroscience, 38 (46), 9925–9933. 10.1523/JNEUROSCI.1429-18.2018 30282726 PMC6234294

[85] Ramirez F., Moscarello J. M., LeDoux J. E., & Sears R. M. (2015). Active avoidance requires a serial basal amygdala to nucleus accumbens shell circuit. Journal of Neuroscience, 35(8), 3470–3477. 25716846 10.1523/JNEUROSCI.1331-14.2015 PMC4339356

[86] Raynolds SM, Berridge KC (2008) Emotional environments retune the valence of appetitive versus fearful functions in nucleus accumbens. Nature Neuroscience 11:423–425 18344996 10.1038/nn2061 PMC2717027

[87] Reppucci C. J., & Petrovich G. D. (2016). Organization of connections between the amygdala, medial prefrontal cortex, and lateral hypothalamus: a single and double retrograde tracing study in rats. Brain structure & function, 221(6), 2937–2962. 10.1007/s00429-015-1081-0 26169110 PMC4713378

[88] Reppucci C. J., & Petrovich G. D. (2018). Neural substrates of fear-induced hypophagia in male and female rats. Brain Structure and Function, 223(6), 2925–2947. doi: 10.1007/s00429-018-1668-3 29704225

[89] Reynolds S. M., & Zahm D. S. (2005). Specificity in the projections of prefrontal and insular cortex to ventral striatopallidum and the extended amygdala. The Journal of neuroscience : the official journal of the Society for Neuroscience, 25(50), 11757–11767. 10.1523/JNEUROSCI.3432-05.2005 16354934 PMC6726011

[90] Salamone J. D. (1994). The involvement of nucleus accumbens dopamine in appetitive and aversive motivation. Behavioural brain research, 61(2), 117–133. 10.1016/0166-4328(94)90153-8 8037860

[91] Saper C.B. (1982), Convergence of autonomic and limbic connections in the insular cortex of the rat. J. Comp. Neurol., 210: 163–173. 7130477 10.1002/cne.902100207

[92] Savander V., Go C.-G., Ledoux J. E., & Pitkänen A. (1995). Intrinsic connections of the Rat Amygdaloid Complex: Projections originating in the basal nucleus. Journal of Comparative Neurology, 361(2), 345–368. 10.1002/cne.903610211 8543667

[93] Sesack S. R., Deutch A. Y., Roth R. H., & Bunney B. S. (1989). Topographical organization of the efferent projections of the medial prefrontal cortex in the rat: an anterograde tract-tracing study with Phaseolus vulgaris leucoagglutinin. The Journal of comparative neurology, 290(2), 213–242. 10.1002/cne.902900205 2592611

[94] Sheynin J., Beck K. D., Pang K. C., Servatius R. J., Shikari S., Ostovich J., & Myers C. E. (2014). Behaviourally inhibited temperament and female sex, two vulnerability factors for anxiety disorders, facilitate conditioned avoidance (also) in humans. Behavioural Processes, 103, 228–235. doi: 10.1016/j.beproc.2014.01.003 24412263 PMC3972301

[95] Shi C. J., & Cassell M. D. (1998). Cortical, thalamic, and amygdaloid connections of the anterior and posterior insular cortices. The Journal of comparative neurology, 399(4), 440–468. 10.1002/(sici)1096-9861(19981005)399:4&lt;440::aid-cne2&gt;3.0.co;2-1 9741477

[96] Stratford TR, Swanson CJ, Kelley A. Specific changes in food intake elicited by blockade or activation of glutamate receptors in the nucleus accumbens shell (1998). Behav Brain Res, 93,1–2, 43–50. doi: 10.1016/s0166-4328(97)00140-x. 9659985

[97] Swanson L. W. (2018). Brain maps 4.0—Structure of the rat brain: An open access atlas with global nervous system nomenclature ontology and flatmaps. Journal of Comparative Neurology, 526(6), 935–943. 10.1002/cne.24381 29277900 PMC5851017

[98] Swanson L. W., & Kohler C. (1986). Anatomical evidence for direct projections from the entorhinal area to the entire cortical mantle in the rat. The Journal of Neuroscience, 6(10), 3010–3023. 10.1523/jneurosci.06-10-03010.1986 3020190 PMC6568776

[99] Swanson L. W., & Petrovich G. D. (1998). What is the amygdala? Trends in Neurosciences, 21(8), 323–331. 9720596 10.1016/s0166-2236(98)01265-x

[100] Terrill SJ, Subramanian KS, Lan R, Liu CM, Cortella AM, Noble E, Kanoski SE (2020) Nucleus accumbens melanin-concentrating hormone signaling promotes feeding in a sex-specifc manner. Neuropharmacology 178:108270 32795460 10.1016/j.neuropharm.2020.108270 PMC7544677

[101] Thompson R. H., & Swanson L. W.. (2010). Hypothesis-driven structural connectivity analysis supports network over hierarchical model of brain architecture. Proceedings of the National Academy of Sciences, 107(34), 15235–15239. 10.1073/pnas.1009112107 PMC2930585 20696892

[102] Treasure J., Duarte T. A., & Schmidt U. (2020). Eating disorders. Lancet (London, England), 395(10227), 899–911. 10.1016/S0140-6736(20)30059-3 32171414

[103] Van Dongen Y. C., Deniau J. M., Pennartz C. M. A., Galis-de Graaf Y., Voorn P., Thierry A. M., & Groenewegen H. J. (2005). Anatomical evidence for direct connections between the shell and core subregions of the rat nucleus accumbens. Neuroscience, 136(4), 1049–1071. 16226842 10.1016/j.neuroscience.2005.08.050

[104] Van Groen T., & Wyss J. M. (1990). Extrinsic projections from area CA1 of the rat hippocampus: Olfactory, cortical, subcortical, and bilateral hippocampal formation projections. The Journal of Comparative Neurology, 302(3), 515–528. 10.1002/cne.903020308 1702115

[105] Vertes R. P. (2002). Analysis of projections from the medial prefrontal cortex to the thalamus in the rat, with emphasis on nucleus reuniens. The Journal of comparative neurology, 442(2), 163–187. 10.1002/cne.10083 11754169

[106] Vertes R. P. (2004). Differential projections of the infralimbic and prelimbic cortex in the rat. Synapse (New York, N.Y.), 51(1), 32–58. 10.1002/syn.10279 14579424

[107] Vertes R. P., & Crane A. M. (1996). Descending projections of the posterior nucleus of the hypothalamus: Phaseolus vulgaris leucoagglutinin analysis in the rat. The Journal of comparative neurology, 374(4), 607–631. 10.1002/(SICI)1096-9861(19961028)374:4&lt;607::AID-CNE9&gt;3.0.CO;2-5 8910738

[108] Vertes RP, Hoover WB (2008) Projections of the paraventricular and paratenial nuclei of the dorsal midline thalamus in the rat. J Comp Neurol 508:212–237. 18311787 10.1002/cne.21679

[109] Wise RA (2000) Interactions between medial prefrontal cortex and meso-limbic components of brain reward circuitry. Progress in Brain Research 126:255–26 DOI: 10.1016/S0079-6123(00)26018-4 11105651

[110] Wright CI, Groenewegen HJ (1996) Patterns of overlap and segregation between insular cortical, intermediodorsal thalamic and basal amygdaloid afferents in the nucleus accumbens of the rat. Neuroscience 73:359–373. doi:10.1016/0306-4522(95)00592-7 8783254

[111] Wu Q. Lemus M.B., Stark R., Bayliss J.A., Reichenbach A., Lockie S.H., Andrews Z.B., (2014). The temporal pattern of cfos activation in hypothalamic, cortical, and brainstem nuclei in response to fasting and refeeding in male mice. Endocrinology, 155 (2014), 840–853 24424063 10.1210/en.2013-1831

[112] Wu Y., Chen C., Chen M., Qian K., Lv X., Wang H., Jiang L., Yu L., Zhuo M., & Qiu S. (2020). The anterior insular cortex unilaterally controls feeding in response to aversive visceral stimuli in mice. Nature Communications, 11(1), 640. 10.1038/s41467-020-14281-5 PMC6994462 32005806

[113] Wyss J.M (1981). An autoradiographic study of the efferent connections of the Entorhinal Cortex in the rat. The Journal of Comparative Neurology, 199(4), 495–512. 10.1002/cne.901990405 6168668

[114] Yu K., Garcia da Silva P., Albeanu D. F., & Li B. (2016). Central amygdala somatostatin neurons gate passive and active defensive behaviors. The Journal of neuroscience: the official journal of the Society for Neuroscience, 36(24), 6488–6496. 10.1523/JNEUROSCI.4419-15.2016 27307236 PMC5015784

[115] Zimmerman J., & Fisher M. (2017). Avoidant/Restrictive Food Intake Disorder (ARFID). Current problems in pediatric and adolescent health care, 47(4), 95–103. 10.1016/j.cppeds.2017.02.005 28532967

